# A Systems-Level Interrogation Identifies Regulators of *Drosophila* Blood Cell Number and Survival

**DOI:** 10.1371/journal.pgen.1005056

**Published:** 2015-03-06

**Authors:** Richelle Sopko, You Bin Lin, Kalpana Makhijani, Brandy Alexander, Norbert Perrimon, Katja Brückner

**Affiliations:** 1 Department of Genetics, Harvard Medical School, Boston, Massachusetts, United States of America; 2 Department of Cell and Tissue Biology, University of California, San Francisco, San Francisco, California, United States of America; 3 Howard Hughes Medical Institute, Boston, Massachusetts, United States of America; 4 Eli and Edythe Broad Center of Regeneration Medicine and Stem Cell Research, University of California, San Francisco, San Francisco, California, United States of America; 5 Cardiovascular Research Institute, University of California, San Francisco, San Francisco, California, United States of America; University of California, Los Angeles, UNITED STATES

## Abstract

In multicellular organisms, cell number is typically determined by a balance of intracellular signals that positively and negatively regulate cell survival and proliferation. Dissecting these signaling networks facilitates the understanding of normal development and tumorigenesis. Here, we study signaling by the *Drosophila* PDGF/VEGF Receptor (Pvr) in embryonic blood cells (hemocytes) and in the related cell line Kc as a model for the requirement of PDGF/VEGF receptors in vertebrate cell survival and proliferation. The system allows the investigation of downstream and parallel signaling networks, based on the ability of Pvr to activate Ras/Erk, Akt/TOR, and yet-uncharacterized signaling pathway/s, which redundantly mediate cell survival and contribute to proliferation. Using Kc cells, we performed a genome wide RNAi screen for regulators of cell number in a sensitized, *Pvr* deficient background. We identified the receptor tyrosine kinase (RTK) *Insulin-like receptor* (*InR*) as a major *Pvr* Enhancer, and the nuclear hormone receptors *Ecdysone receptor* (*EcR*) and *ultraspiracle* (*usp*), corresponding to mammalian Retinoid X Receptor (RXR), as *Pvr* Suppressors. *In vivo* analysis in the *Drosophila* embryo revealed a previously unrecognized role for EcR to promote apoptotic death of embryonic blood cells, which is balanced with pro-survival signaling by Pvr and InR. Phosphoproteomic analysis demonstrates distinct modes of cell number regulation by EcR and RTK signaling. We define common phosphorylation targets of Pvr and InR that include regulators of cell survival, and unique targets responsible for specialized receptor functions. Interestingly, our analysis reveals that the selection of phosphorylation targets by signaling receptors shows qualitative changes depending on the signaling status of the cell, which may have wide-reaching implications for other cell regulatory systems.

## Author Summary

Signaling networks that drive cell survival and proliferation regulate cell number in development and disease. We use a simple *Drosophila* model of cell number control, which centers on PDGF/VEGF receptor signaling. Performing a genome-wide RNAi screen under Pvr-sensitized conditions, we identify regulators of cell number that have not been found in conventional screens. Validation by in vivo genetics reveals previously unrecognized roles for EcR and InR in the balance of cell survival in the *Drosophila* embryo. Phosphoproteomic analysis demonstrates distinct mechanisms of cell survival regulation by EcR and receptor tyrosine kinase signaling. It further identifies common phosphorylation targets of Pvr and InR including regulators of cell survival, and receptor-specific phosphorylation targets mediating unique functions of Pvr and InR. Importantly, the study provides precedence that the selection of phosphorylation targets by signaling receptors can change with the signaling status of the cell, which may have wide-reaching implications for other cell regulatory systems.

## Introduction

The regulation of cell number varies greatly and typically depends on developmental and environmental stimuli that determine the intracellular balance of pro- and anti-death, and proliferative signals [1–3]. Proto-oncogenes and tumor suppressors play roles as regulators of cell number and the pathological extension of cell survival is a major hallmark of tumorigenesis [4]. Accordingly, understanding the complex signaling networks that regulate cell survival is an important yet incompletely accomplished goal [4,5], which can be facilitated by studying a simple model organism.

Blood cells in the fruitfly *Drosophila melanogaster* have been instrumental in the discovery of fundamental concepts in immunity, hematopoiesis and wound healing [6–11], but they are also a convenient model to study mechanisms that regulate cell number. In particular, the *Drosophila* PDGF/VEGF Receptor (Pvr), a member of the Receptor Tyrosine Kinase (RTK) family, controls anti-apoptotic survival signaling in *Drosophila* blood cells (hemocytes) *in vivo* and in the embryonic cell line Kc in culture [12]. In other instances, Pvr has been reported to regulate cell proliferation [13,14], differentiation [15,16], cell size [17,18], cytoskeletal architecture [19] and cell migration [20–22]. *Drosophila* Pvr therefore parallels roles of the vertebrate family of PDGF/VEGF Receptors in development and disease [12,21,23–26].

Here, we took advantage of the role of Pvr in embryonic blood cell survival and performed a systematic RNAi screen to identify regulators of cell number, using the *Drosophila* cell line Kc under sensitized conditions of *Pvr* knockdown. The screen identified enhancers and suppressors of the *Pvr* RNAi phenotype, many of which were not found in conventional RNAi screens examining cell growth and viability. In particular, we found that knockdown of *InR* enhanced the *Pvr* RNAi phenotype while knockdown of the *Ecdysone receptor* (*EcR*) [27] and its co-receptor *ultraspiracle* (*usp*) [28] suppressed the *Pvr* RNAi phenotype. We confirmed functional roles for these genes related to Pvr both in cell culture and *in vivo*. Phosphoproteomic analyses revealed major differences in the signaling signature of *Pvr* deficient cells rescued by activation of InR as compared to inactivation of EcR. Further, our analysis identified distinct sets of phosphorylation targets, common to both Pvr and InR, and unique to each receptor. Most importantly, we provide precedence that the selection of phosphorylation targets by signaling receptors can depend on the signaling status of the cell, which may have wide-reaching implications for cell regulatory systems in animal development, disease, and the experimental and therapeutic manipulation of signaling pathways.

## Results

### Pvr signaling in the embryonic hemocyte cell line Kc

Previously, we demonstrated that the *Drosophila* PDGF/VEGF Receptor, Pvr, is essential for anti-apoptotic survival in embryonic hemocytes and in the related cell line Kc, which maintains autocrine Pvr signaling [12,29]. Taking advantage of these systems, we sought to examine the signaling networks that mediate anti-apoptotic survival and regulate cell number.

**Fig 1:**
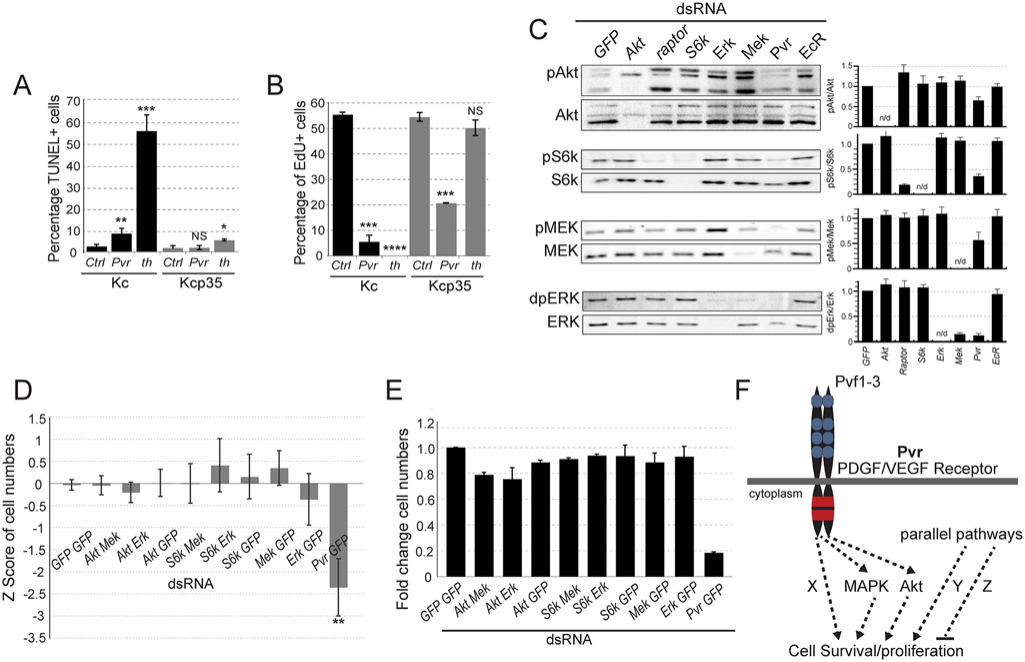
Signaling pathways in Pvr survival signaling. (A, B) Comparison of Kc and p35-expressing Kcp35 cells, studying the effects of Pvr RNAi silencing on cell death and cell proliferation. (A) dsRNA treatment targeting *Pvr* or *thread* (*th*) (positive control) causes increased fractions of cells undergoing apoptosis, measured by TUNEL assay. As expected, apoptosis is largely suppressed in the presence of p35 (Kcp35 cells). (B) EdU proliferation assay in Kc and Kcp35 cells. Suppression of programmed cell death by p35 reveals that Pvr silencing results in reduced proliferation. (C) Representative immunoblots of phospho-mediators of the Mek/Erk and Akt/Tor pathways. RNAi silencing of genes as indicated in the top panel. Note that *Pvr* knockdown leads to reduced activities of both pathways. The bar graphs (right) represent ratios of quantified phosphoprotein signal to total protein signal, relative to GFP control knockdown. The data are representative of three independent experiments. (D, E) Combined knockdown of Akt/Tor and Mek/Erk pathway components (as indicated) does not match the effect of *Pvr* silencing, suggesting additional, redundant survival pathways downstream of Pvr. (D) CellTiter-Glo assay measuring ATP concentration as a readout of cell number. Z score relative to all samples is shown. (E) Cell counts in an assay corresponding to (D). (F) Hypothesis of Pvr-mediated survival and proliferation signaling. Pvr survival signaling relies on several redundant pathways (Akt, Mapk, Pathway X). In addition, parallel pro-and anti-death pathways (Pathway Y, Z) are proposed. In Pvr loss of function conditions, selective re-activation of an anti-apoptotic pathway by silencing of a suppressor, or inhibition of a pro-apoptotic regulator, suffices to restore cell numbers.

First, we confirmed that RNAi-mediated knockdown of *Pvr* induces apoptotic cell death in Kc cells. RNAi silencing of the *Drosophila* inhibitor of apoptosis *DIAP1*, or *thread* (*th*), served as positive control (Fig. 1A). Expression of the baculovirus inhibitor of apoptosis *p35* [30] rescued hemocyte survival, leading us to establish a selected pool of Kcp35 cells (Kcp35 cells, Fig. 1A). Immunoblotting confirmed that Pvr knockdown was equally efficient in Kc and Kcp35 cells (S1 Fig). Closer examination by incorporation of the thymidine nucleoside analog EdU (5-ethynyl-2’deoxyuridine) in Kc versus Kcp35 cells revealed that *Pvr* also moderately contributes toward cell proliferation in this system (Fig. 1B), an effect that could not be distinguished in a previous study employing cell cycle profiling [12]. Reduction in proliferation was also suggested by immunoblotting, where lysates of equal numbers of cells showed a decrease in the proliferation marker phospho-histone H3 (pHH3) in *Pvr* knockdown samples (S1 Fig).

Using Kc cells, we queried signaling pathways that might be involved in *Pvr*-dependent cell survival and proliferation. Examining activity of the Akt/TOR and Mek/Erk pathways by using antibodies to phosphorylated forms of S6Kinase (S6K, an Akt pathway target), Mek and Erk, we found that both pathways are active in Kc cells. *Pvr* RNAi led to a significant reduction in the phosphorylation levels of these proteins, indicating that Pvr is a major activator of these pathways in Kc cells. Single signaling mediator knockdowns of Akt, the TOR-associated Raptor, S6K, Mek and Erk served as controls (Fig. 1C). Phosphorylation signals were also quantified and displayed as a ratio with the amount of unphosphorylated signaling mediator (Fig. 1C). These findings suggest that Pvr triggers activation of the Akt/TOR and Mek/Erk and pathways, thereby supporting anti-apoptotic cell survival and proliferation.

Next, we asked whether silencing of either or both of these pathways is sufficient to affect cell viability and mimic loss of *Pvr* function. Combining dsRNAs targeting various mediators of the Akt/Tor and Mek/Erk pathways, we found that, despite efficient knockdown of the genes (S2 Fig), neither single nor simultaneous inhibition of both pathways caused a significant reduction of cell numbers, as quantified by CellTiterGlo assay based on ATP content (Fig. 1D), and cell counting (Fig. 1E). In contrast, *Pvr* RNAi, showed significant decreases in cell number (Fig. 1D, E). This predicted the presence of one or more additional, redundant cell survival/proliferation pathway(s) downstream of Pvr (‘X’, Fig. 1F), and/or parallel signaling pathways that contribute to the overall survival and proliferation of the cell (‘Y’, Fig. 1F).

### A genome-wide RNAi screen for modifiers of *Pvr*

**Fig 2:**
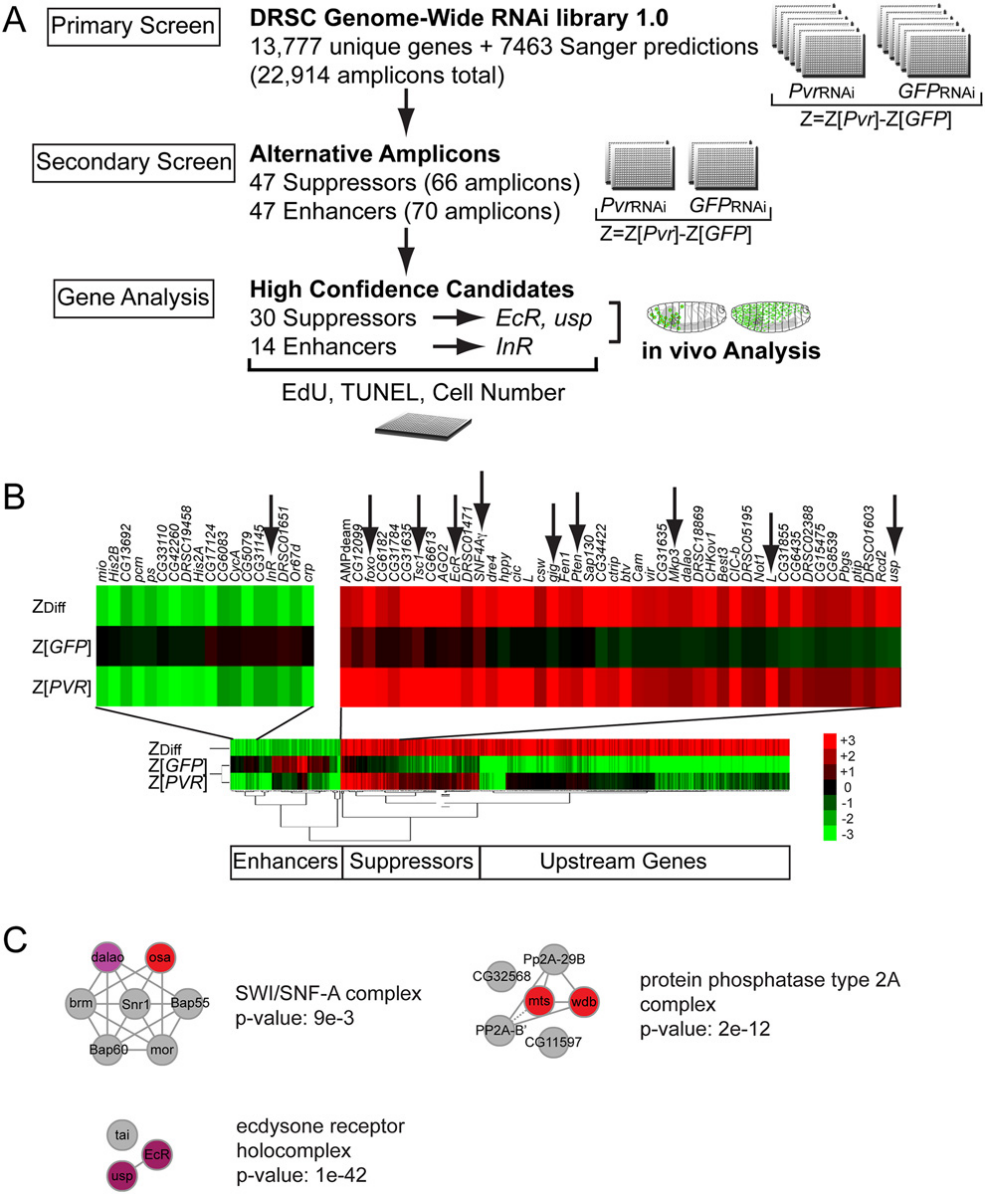
Genome-wide RNAi Pvr modifier screen. (A) Screen scheme of the Primary and Secondary Screens, and subsequent single gene analysis. (B) Cluster analysis of primary screen hits, highlighting a fraction of *Pvr* Enhancers including InR (arrow) and *Pvr* Suppressors including *EcR* and *usp* (arrows). (C) Protein complexes enriched in the list of *Pvr* Suppressors from the RNAi screen, identified using COMPLEAT [154]. Node color correlates with phenotypic strength where red is strongest and purple is weakest, while gray nodes were not identified in the screen. Note p-values are approximate given that the random proteins used to generate the p-value are unique for each of 1000 random sets.

Based on our prediction, we sought to identify other signaling pathways that contribute to the anti-apoptotic survival of Kc cells. We hypothesized that re-activation of just one survival or proliferation pathway would be sufficient to rescue cell numbers in *Pvr* deficient cells (Fig. 1F). Indeed, silencing of negative regulators of the Akt/Tor and Erk pathways rescued the *Pvr* RNAi phenotype, validating our screening approach. For these experiments, we ruled out that silencing of downstream signaling mediators such as Akt would result in upregulation of *Pvf2 expression*, the major Pvr ligand in Kc cells that mediates autocrine signaling (S3 Fig). Expanding our approach, we screened the DRSC Genome-Wide RNAi library 1.0 (*Drosophila* RNAi Screening Center, Harvard Medical School) for modifiers of cell number, specifically under conditions of *Pvr* RNAi-mediated silencing compared to a control background (Fig. 2A). The DRSC 1.0 set targets 22,914 distinct amplicons based on Flybase release 5.51 of the *Drosophila* genome, corresponding to 13,777 unique genes [31], 6944 of which are expressed in Kc cells [29]. Screening was performed in 384-well format, quantifying ATP content as a readout of cell number (CellTiterGlo). To determine an increase or decrease over the average value of ATP content, Z scores were calculated for each well. Focusing on those dsRNAs that show differential effects in *Pvr* knockdown (*Pvr* RNAi) versus control cells (*GFP* RNAi), we calculated the difference of each of the Z scores (ZDiff = Z[*Pvr*]-Z[*GFP*]), and selected amplicons with ZDiff> = 2 and ZDiff< = -2 as primary screen hits (S1 Table). Cluster analysis of the values Z[*Pvr*], Z[*GFP*], and ZDiff for each amplicon revealed three distinct classes of signatures, i.e. *Pvr* Suppressors, *Pvr* Enhancers, and *Pvr* ‘Upstream Genes’ (Fig. 2B). By our cutoff criteria, 64 amplicons scored as suppressors of the *Pvr* knockdown phenotype, rescuing cell numbers more effectively in the *Pvr* RNAi background compared to control cells. 65 amplicons scored as *Pvr* Enhancers, exacerbating the *Pvr* knockdown phenotype. We classified 290 amplicons as *Pvr* ‘Upstream Genes’, reducing cell numbers in control cells, but having rather minor effects in the *Pvr* silenced background. Among this group we found amplicons targeting *Pvr* itself and many ribosomal proteins, suggesting that many of the targeted genes play a role in the production or activation of Pvr (S1 Table).

Subsequent secondary testing of screen hits was carried out for *Pvr* Suppressors and *Pvr* Enhancers. We selected 47 suppressor genes and 47 enhancer genes based on a cutoff of ZDiff> = 2.2 and ZDiff< = -2.2 (S2 Table) and synthesized non-overlapping alternative amplicons that were free of 19bp or larger overlaps with other genes, in order to avoid off-target effects [32,33]. As in the primary screen, amplicons were tested for their ability to modify cell number, specifically comparing *Pvr* knockdown cells relative to control cells (S2 Table). To identify promising ‘high confidence candidates’ for further analysis, we calculated the average of the ZDiff scores among all amplicons of a gene from the primary and secondary screens (ZDiffFinal) (S3 Table). Based on ZDiffFinal values of > = 1.6 and < = -1.2, we report 30 high-confidence *Pvr* Suppressors and 14 high-confidence *Pvr* Enhancers (S3 Table). Z value cutoffs were guided by the scores of predicted genes within the set, such as members of the Akt/Tor and Mek/Erk pathways. Candidates of specific interest were confirmed by live/dead cell counting, omitting genes with obvious roles in RNA interference, such as AGO2 (S4 Fig).

Relatively few genes scored as *Pvr* Enhancers. Among those, we identified the RTK *InR* [34], and *cropped* (*crp*) encoding the helix-loop-helix transcription factor that is a homolog of the mammalian transcription factor AP-4 [35]. The screen also identified *tonalli* (*tna)*, encoding a protein similar to mammalian ZMIZ1 and ZMIZ2 involved in sumoylation [36] that interacts genetically with the Brahma ATP-dependent chromatin remodeling complex in *Drosophila* [37].

Among the *Pvr* Suppressors, the screen yielded all known tumor suppressors and negative regulators of the Akt/TOR pathway, including *Phosphatase and Tensin Homolog* (*Pten)*, *Tuberous Sclerosis Protein 1* (*Tsc1*), *gigas* (*gig*)/*Tuberous Sclerosis Protein 2* (*Tsc2*), *SNF4A–a and -γ*, also known as *AMP-Activated Protein Kinase subunits a and γ(AMPK–α* and *AMPK–γ*), *Forkhead Box Protein* (*foxo*), and *Lobe* (*L*), a protein with similarities to the vertebrate *Proline-rich Akt substrate of 40 kDa* (*PRAS40*) [38–41]. We further identified negative regulators of the Ras/Erk pathway *Mitogen-activated protein kinase phosphatase 3* (*Mkp3*), and *microtubule star* (*mts*) and *widerborst* (*wdb*), which encode components of the protein phosphatase PP2A complex [42–44]. We calculated which protein complexes were over-represented with respect to the frequency of their components among the high confidence hits in the RNAi screen, and found, besides the PP2A complex, two other major protein complexes among the high confidence hits in the RNAi screen (Fig. 2C): the ecdysone receptor complex, consisting of the nuclear hormone receptors *EcR* and *usp* [27,45], and the Brahma SWI2/SNF2 family ATPase chromatin-remodeling complex, comprising *osa* and *dalao* [46,47]. Other Pvr Suppressors were *CG6182*, an ortholog of mammalian *TBC1 domain member 7* (*TBC7)*, and *GckIII*, and *CG31635*, an ortholog of mammalian *LRRC68*. Given the reported interplay between ecdysone and insulin signaling during development [48], we wanted to dissect whether common and/or distinct downstream mechanisms mediate Pvr suppression, and therefore chose InR and *EcR* /*usp* for in vivo validation.

### Pro-apoptotic effects of EcR/Usp signaling and anti-apoptotic effects of insulin signaling in cell culture

**Fig 3:**
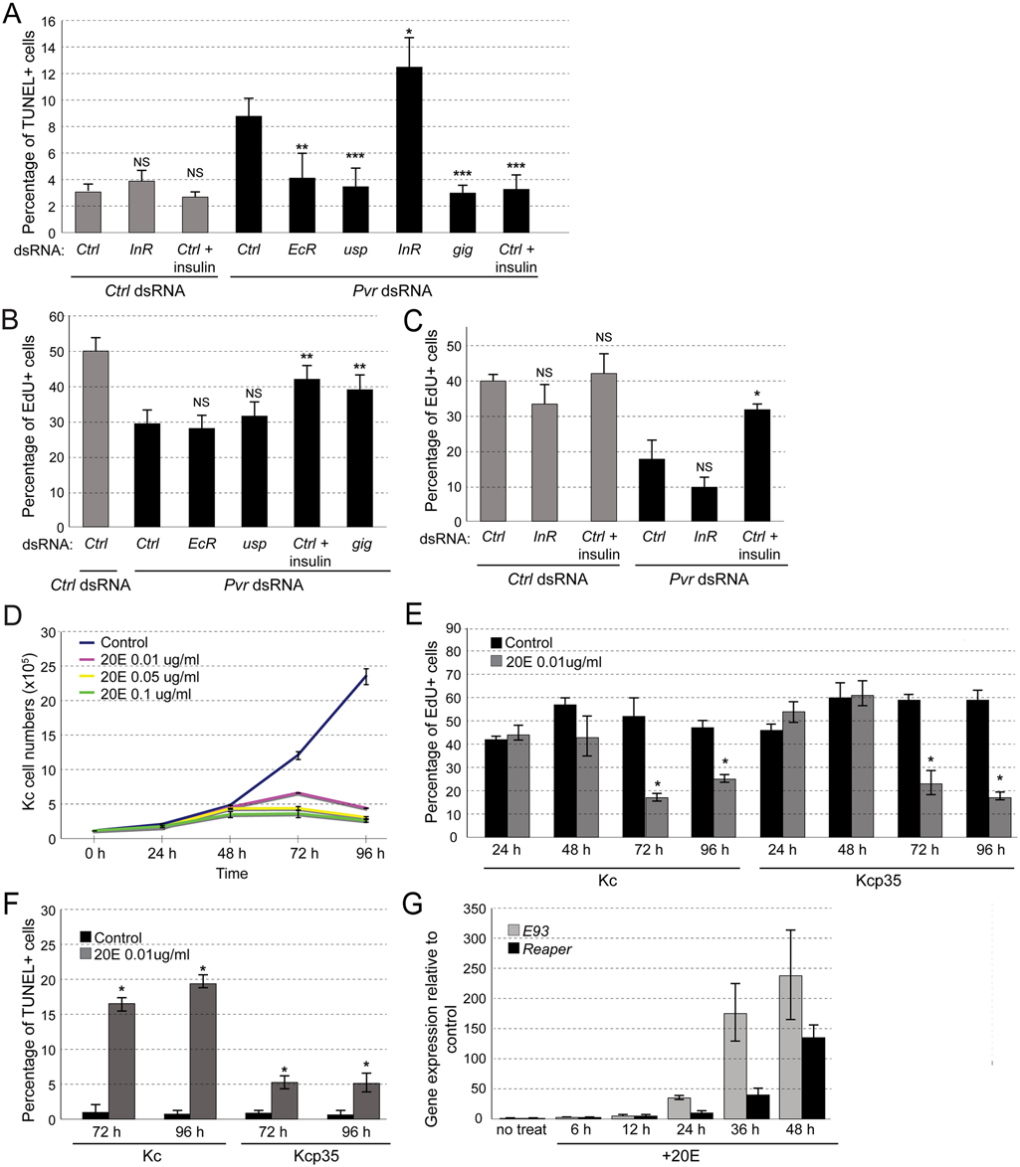
Effects of EcR and insulin signaling in Kc cells. (A) Effects of InR silencing and activation, and EcR silencing on *Pvr* knockdown-induced cell death. TUNEL assay in Kc cells and quantification of percentage of TUNEL positive cells. (B, C) EdU incorporation assay in Kcp35 cells to quantify rescue of proliferation defects in the Pvr silenced background. (B) InR stimulation by addition of insulin, or re-activation of the Akt/Tor pathway by *gig* knockdown rescue proliferation in *Pvr* RNAi cells; silencing of *EcR* or *usp* do not show significant effects. (C) Silencing of InR insignificantly reduces cell proliferation in the Pvr kd and control background. InR stimulation by addition of insulin rescues proliferation in *Pvr* RNAi cells significantly. (D) Effect of ecdysone (20E) treatment on Kc cell numbers over time. 20E concentrations as indicated. (E) EdU incorporation in Kc and Kcp35 cells, treated with 20E or control solvent. Time points and concentration as indicated. (F) Percentage of TUNEL positive cells in Kc and Kcp35, treated with 20E or control solvent for the indicated times. (G) Upregulation of *rpr* and *E93* upon stimulation with 20E in Kc cells, as determined by qRT-PCR.

Using Kc cells, we examined the functional roles of InR and EcR/Usp in more detail. EcR and Usp form a heterodimer and are induced by binding of the steroid hormone 20-hydroxyecdysone (20E) [45,49]. Signaling by the EcR complex plays a major role during molting and metamorphosis [50], yet a role in embryonic cell death and cell number control has not been established [51]. We confirmed the effects of silencing or stimulating InR, or silencing *EcR* or *usp*, on *Pvr* RNAi-induced apoptosis using TUNEL assays, and we quantified effects on proliferation using EdU incorporation in Kcp35 cells (Fig. 3A-C). Consistent with the results from the screen, we found that, in combination with *Pvr* knockdown, silencing of *InR* exacerbated apoptosis. Further, silencing of *EcR* or *usp*, or stimulation of InR with insulin rescued apoptosis (Fig. 3A). In contrast, when examining proliferation, only insulin stimulation or a Tsc2/gigas (gig) RNAi Akt pathway control significantly suppressed proliferation defects, suggesting that EcR and Usp mainly function in the regulation of cell death, rather than proliferation (Fig. 3B, C). InR knockdown seemed to enhance the reduction of EdU incorporation in Pvr knockdown cells, but differences were not statistically significant based on three independent biological replicate experiments (Fig. 3C).

Next we examined the effects of ecdysone stimulation. Anti-proliferative effects of ecdysone in Kc cells have been reported previously [52–54], but whether ecdysone also has direct pro-apoptotic effects in embryonic cells not been determined. To test this, we stimulated Kc cells with 20E at concentrations close to physiological levels (0.01ug/ml) [54,55]. Overall, 20E induced a marked reduction in cell number at stimulation times of >48h (Fig. 3D). As expected, it resulted in a reduction of cell proliferation as measured by EdU incorporation, both in Kc and in apoptosis-resistant Kcp35 cells (Fig. 3E). However, TUNEL analysis showed a substantial increase in apoptotic cells upon 20E stimulation, which was largely suppressed in Kcp35 cells (Fig. 3F). 20E did not cause a decrease of Pvr protein levels (S5 Fig), suggesting that molecular mechanisms other than Pvr downregulation account for the observed increase in apoptosis. During metamorphosis-associated programmed cell death (PCD), several genes have been described as ecdysone-induced pro-death targets, in particular *Ecdysone-induced protein 93F* (*E93)*, *broad* (*br*), *Ecdysone-induced protein 74EF* (*E74A*), and *reaper* (*rpr*) [56–58]. When we examined the expression levels of these genes during ecdysone stimulation of Kc cells we found that, indeed, *rpr* and *E93* levels increased from the first day of 20E stimulation (Fig. 3G), consistent with the induction of apoptosis. We also examined whether Pvr knockdown would have an effect on the expression of *rpr* and *E93* but found no significant difference relative to controls (S6 Fig).

In summary, we conclude that the EcR complex has pro-apoptotic functions in the cell line Kc, which become apparent under sensitized conditions of *Pvr* loss of function, or experimental addition of 20E.

### EcR and InR are opposing modifiers of Pvr *in vivo*

**Fig 4:**
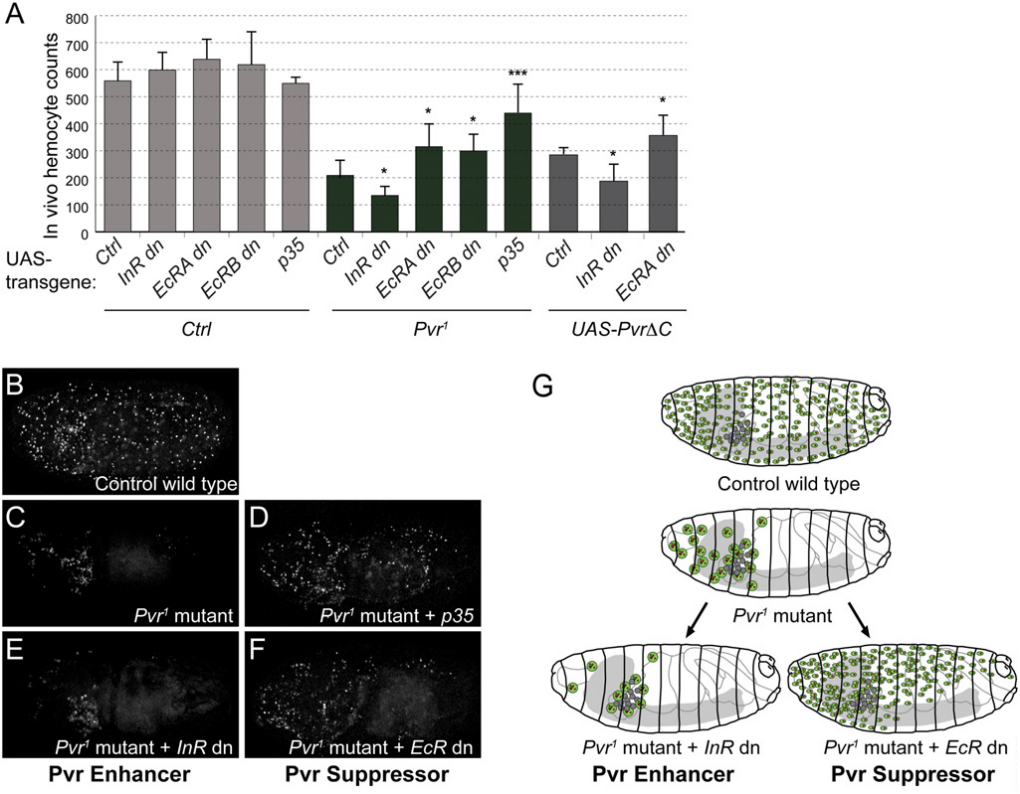
Role of EcR and InR in embryonic hemocytes *in vivo*. (A) In vivo rescue experiment, combining Pvr1 mutant or hemocyte specific expression of UAS-PvrΔC with various UAS-transgenes. Inhibition of EcR signaling by dominant-negative EcR (EcRA dn or EcRB dn) rescues hemocyte numbers in *Pvr* ^*1*^ mutant embryos, while co-expression of dominant-negative InR enhances the *Pvr* ^*1*^ phenotype. Embryonic hemocyte numbers in embryos of the indicated genetic combinations. Hemocytes were marked by nuclear β-Gal driven by srpHemoGAL4; total hemocytes of individual stage 16 embryos were counted. For full genotype, see Methods. (B-F) Confocal images of representative embryos. (G) Summary model of Pvr Suppressors and Pvr Enhancers as shown in B-F. In Pvr1 mutants, apoptotic hemocytes are phagocytosed by remaining, viable hemocytes, which increase in size [12]. Additional lack of a Pvr Enhancer aggravates the phenotype, while lack of a Pvr Suppressor rescues hemocyte death.

To test the role of *InR* and *EcR* in the suppression and enhancement of apoptosis *in vivo*, we examined the function of these genes in the survival of hemocytes in the *Drosophila* embryo. *Drosophila* embryos typically show a developmentally fixed number of ~600 hemocytes post stage 11 until early stage 17, and loss of Pvr signaling causes a rapid decline in hemocytes due to their apoptotic death and phagocytic clearance by the small number of remaining live hemocytes [12]. Based on our findings in Kc cells, we predicted that inhibition of EcR would rescue, and inhibition of InR would enhance, *Pvr* loss-of-function in embryonic blood cells [12]. Indeed, hemocyte-specific suppression of EcR signaling by expression of dominant-negative forms of EcR [59,60] partially rescued hemocyte counts in *Pvr* ^*1*^ mutant embryos (Fig. 4), resembling rescue by the baculovirus inhibitor of apoptosis, p35 [12] (see also Fig. 4A). Conversely, expression of dominant-negative InR in hemocytes enhanced the *Pvr* phenotype, further reducing embryonic hemocyte numbers (Fig. 4). Consistently, we previously demonstrated that activated PI3K, a positive mediator of the Akt/TOR pathway downstream of InR, can partially rescue the *Pvr* mutant in vivo phenotype [12]. To confirm hemocyte autonomous effects of EcR and InR, we induced embryonic hemocyte death by hemocyte-specific expression of dominant-negative PvrΔC [12], and examined the effects of co-expressed dominant-negative versions of EcR or InR. Again, we found that dominant-negative EcR rescued apoptotic loss of hemocytes, while dominant-negative InR exacerbated the cell death phenotype (Fig. 4). Expression of the transgenes in the wild type background had no significant effects (Fig. 4A).

Intrigued by the mild increase of hemocyte numbers upon overexpression of dominant-negative EcRdn (Fig. 4A), we asked whether blocking EcR signaling alone would have a positive effect on hemocyte numbers at a later point during development, for example at the transition from the embryo to the larval stage. A time course of total hemocyte counts in live animals illustrates that, compared to stage 16 embryos, hemocyte numbers in young 1^st^ instar larvae decline to about 60%, suggesting a putative connection with the embryonic ecdysone peak in mid-embryogenesis [61] (S7 Fig). However, comparing live hemocyte counts of controls to animals with hemocyte-specific expression of EcRdn, we did not see a significant rescue in the total number of hemocytes, despite a mild increase in EcRdn overexpressing larvae (S7 Fig).

Taken together, our findings suggest that EcR signaling accounts for a basic level of pro-death signaling in embryonic hemocytes, which however is revealed only under sensitized conditions such as *Pvr* loss of function. Conversely, signaling by InR contributes to the trophic survival of embryonic hemocytes, which acts redundantly with Pvr signaling, and therefore again is only evident in conjunction with loss of Pvr signaling (Fig. 4G).

### Signaling networks downstream of Pvr, InR and EcR

**Fig 5:**
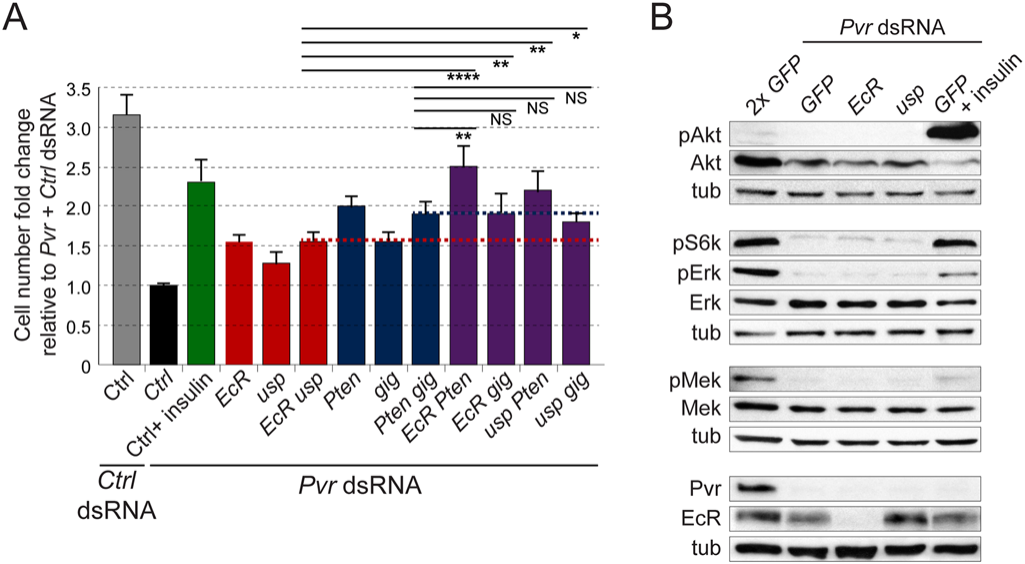
Crosstalk between RTK and EcR signaling. (A) Effects of insulin stimulation or gene silencing on the cell number of *Pvr* silenced Kc cells. Note additive effects of knockdown of combinations of genes from the Akt and EcR pathways in the rescue of *Pvr* deficient cell numbers (bars above red or blue lines, respectively). In all cases, total amounts of dsRNA were adjusted by addition of *GFP* control dsRNA. (B) Immunoblot for phosphorylated signaling mediators in *Pvr* silenced Kc cells under the indicated stimulation/knockdown conditions. Note that insulin stimulation rescues Akt and S6K phosphorylation in the *Pvr* RNAi background, while *EcR* RNAi and *usp* RNAi have no effect.

Based on our findings, we sought to further dissect the relationship between Pvr, InR and EcR signaling. First, we asked whether signaling by the EcR complex acts epistatically or in parallel with RTK-triggered signaling pathways such as Akt/Tor. When comparing the effects of silencing of the EcR/Usp and Akt/Tor pathways separately and in combination, we found that simultaneous knockdown of genes from both pathways resulted in increased cell number rescue (*e*.*g*. *EcR* and *Pten*), which in many cases was significant when compared to knockdown of two genes from the same pathway (*i*.*e*. *EcR* and *usp*, *or Pten and gig*). This suggested a parallel, rather than epistatic relationship (Fig. 5A). Biochemically, insulin stimulation of *Pvr* deficient cells restored, albeit to distinctive levels, phosphorylation of downstream signaling mediators of the Akt/Tor and Mek/Erk pathways, while EcR knockdown did not show such effects (Fig. 5B). This suggested similar but not identical signaling profiles for the RTKs Pvr and InR, and distinct mechanisms for the EcR complex.

To compare the signaling profiles of Pvr, InR and EcR in a more systematic manner, we chose a phosphoproteomics approach. We utilized mass spectrometry and an isobaric labeling strategy that enables multiplexing and relative quantification between samples [62,63]. For this analysis, we surveyed the phosphoproteome by formally comparing conditions of (1) ‘high Pvr’ signaling (+ control dsRNA; taking advantage of the high endocrine Pvr activity in Kc cells); (2) ‘low Pvr’ signaling (+ Pvr dsRNA); (3) ‘high InR’ signaling (+ insulin, to stimulate endogenous InR in Kc cells); (4) ‘low InR’ signaling (+ control dsRNA; taking advantage of the low InR activity in Kc cells under standard culture conditions presumably due to low levels of dIlp expression [29], see also Fig. 5B); (5) ‘high EcR’ (endogenous EcR in Kc cells); and (6) ‘low EcR’ (+ EcR dsRNA).

**Fig 6:**
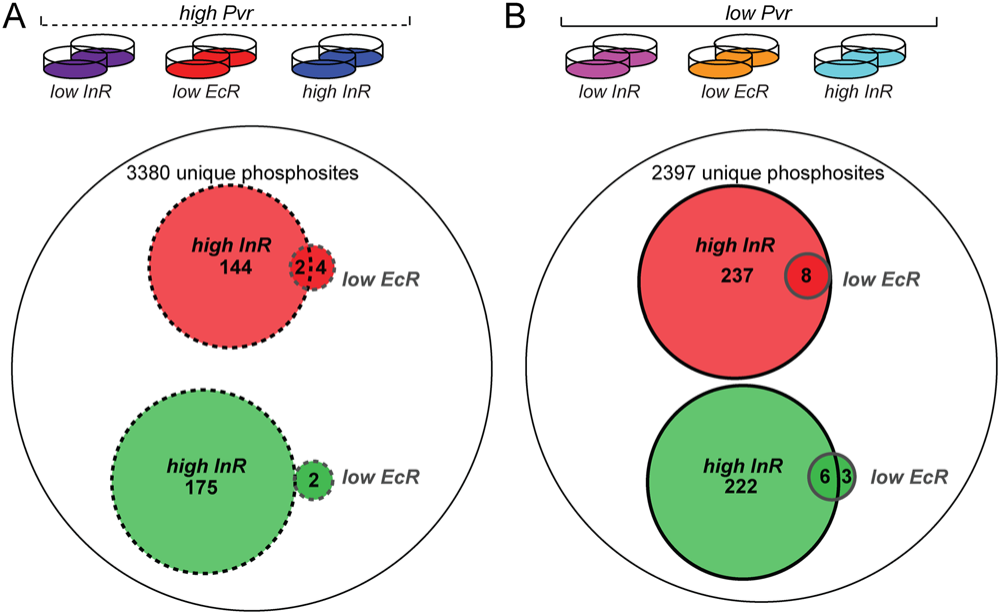
Phosphoproteome survey of Kc cells under *Pvr* rescue conditions. (A) From left to right the conditions for Kc cells subjected to phosphopeptide enrichment and mass spectrometry were ‘high PVR, low InR’, ‘high PVR, low EcR, and ‘high PVR, high InR’. Of 3380 unique phosphosites identified by mass spectrometry in both replicates, 144 phosphosites were upregulated on average >1.5-fold (red) in response to insulin while 176 phosphosites were downregulated on average >1.5-fold (green). Knockdown of *EcR* induced more than 1.5-fold up- and down-regulation of 6 and 2 phosphosites, respectively. (B) From left to right the conditions for Kc cells subjected to phosphopeptide enrichment and mass spectrometry were ‘low PVR, low InR’, ‘low PVR, low EcR, and ‘low PVR, high InR’. Of 2397 unique phosphosites identified in both replicates, 237 phosphosites were upregulated on average >1.5-fold in response to insulin while 222 phosphosites were downregulated on average >1.5-fold in Pvr RNAi cells. *EcR* knockdown induced more than 1.5-fold up- and down-regulation of 8 and 9 phosphosites, respectively in PVR RNAi cells.

First, we assessed which phosphoproteins were up- or downregulated in the rescue of *Pvr* silenced Kc cells. We surveyed the phosphoproteome under conditions of high and low Pvr activity (Fig. 6A and B, respectively), and analyzed separately for the two conditions the effects of EcR silencing or InR activation, assessing biological duplicates (S4 Table and S5 Table). Under ‘high Pvr’ conditions, approximately 10% of the detected phosphorylation was altered more than 1.5-fold under conditions of InR stimulation, which we refer to as the ‘InR-specific set’ (Fig. 6A and S4 Table). This percentage nearly doubled under ‘low Pvr’ conditions (Fig. 6B and S5 Table). Although some of these phosphorylations could be attributed to the fact the InR may phosphorylate Pvr targets in the absence of Pvr, this finding also suggested the emergence of new sets of up and down-regulated phosphosites that were not observed upon InR activation under ‘high Pvr’ conditions (below).

**Fig 7:**
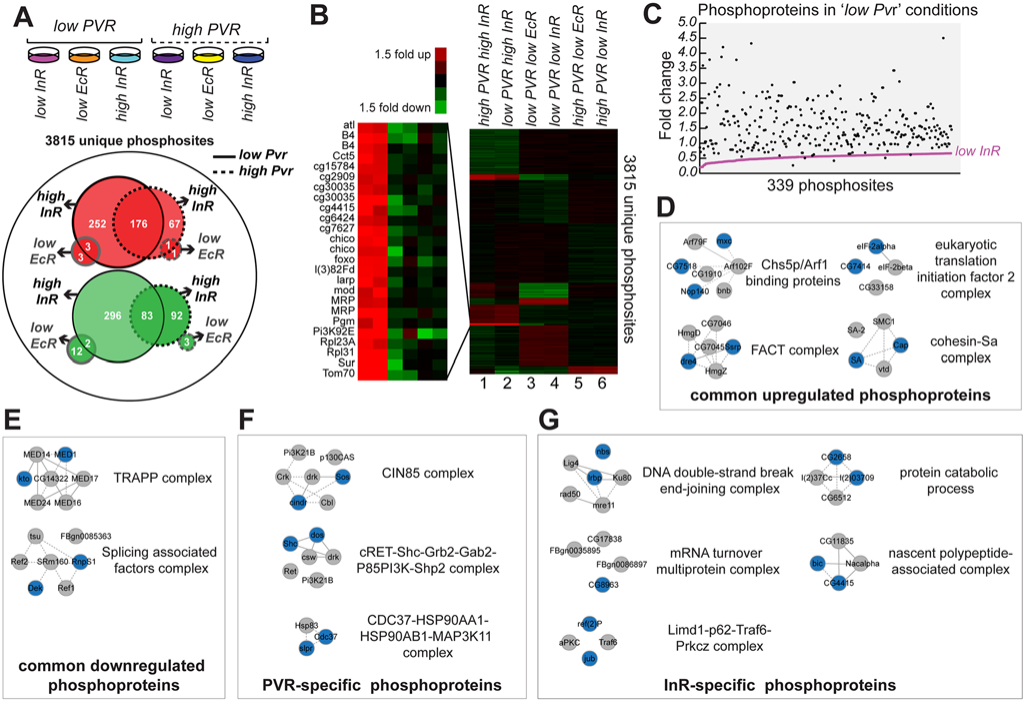
Relative phosphopeptide quantitation comparing ‘low *Pvr’* and ‘high Pvr’ conditions. (A) From left to right conditions the conditions for Kc cells subjected to phosphopeptide enrichment and mass spectrometry were: ‘low Pvr, low InR’, ‘low Pvr, low EcR’, ‘low Pvr, high InR’, ‘high Pvr, low InR’, ‘high Pvr, low EcR’ and ‘high Pvr, high InR’. Of 3815 unique phosphosites identified, 176 phosphosites were upregulated >1.5-fold (red) in response to insulin treatment both in ‘low Pvr’ and ‘high Pvr conditions’ while 83 phosphosites were commonly downregulated >1.5-fold (green). *EcR* RNAi resulted in only one phosphosite upregulated >1.5-fold in both ‘low Pvr’ and ‘high Pvr’ conditions while two phosphosites were downregulated >1.5-fold in both conditions. More phosphosites were found changing in *‘low Pvr’* versus ‘high Pvr’ conditions (solid versus dashed lines), both in response to insulin and *EcR* RNAi (black versus grey lines). (B) Dendogram of identified phosphoproteome from A, normalized to the median change for each phosphopeptide identified and clustered using k-means analysis (K = 10 gene clusters) and Euclidean distance as a similarity measure. (C) Insulin stimulation restores phosphorylation to nearly all of 339 phosphosites downregulated >1.5-fold in ‘low Pvr’ conditions relative to ‘high Pvr’ conditions (S6 Table: ‘Pvr-specific set’). (D) Complexes enriched for components (colored nodes) for which phosphorylation is targeted by both Pvr and InR. (E) Complexes enriched for components (colored nodes) for which phosphorylation is downregulated when Pvr and InR are active. (F) Complexes enriched for components (colored nodes) specifically regulated by Pvr. (G) Complexes enriched for components (colored nodes) for which phosphophorylation is specifically regulated by InR.

Secondly, we sought to directly measure the degree to which phosphosites were altered by InR under conditions of ‘high’ versus ‘low’ Pvr signaling, hypothesizing a ‘sensitization’ of InR signaling by the absence of *Pvr*. We repeated our phosphoproteomic analysis, this time directly comparing the six experimental signaling conditions among each other (Fig. 7A and S6 Table). While nearly three-quarters of the ‘InR-specific set’ of phosphopeptides remained upregulated following InR activation in the absence of *Pvr*, the ‘InR-specific set’ showed qualitative differences in the absence and presence of Pvr signaling. For instance, InR stimulation elevated levels of phosphorylation of fifteen phosphoproteins specifically under ‘low Pvr’ activity as compared to ‘high Pvr’ signaling. These included Chromosome-associated protein (Cap), lava lamp (lva), Enhancer of decapping 3 (Edc3), Bicaudal D (BicD), lethal(2)03709, eukaryotic translation Initiation Factor 2α (eIF-2 α) and several uncharacterized gene products. InR activation restored phosphorylation to nearly all sites downregulated in *Pvr* deficient cells, (Fig. 7C). These phosphorylations likely account for the ability of insulin to rescue *Pvr* deficiency. *EcR* knockdown, meanwhile, had very little effect on the phosphoproteome, both in low and high *Pvr* conditions (Fig. 6A, B), despite efficient knockdown (S8 Fig). Similar findings were made from the comparative analysis of all six experimental conditions (Fig. 7A, B). This is consistent with an alternative mode of action, such as the transcriptional modulation of EcR/Usp target genes (see Fig. 3G).

Lastly, we identified a pool of common phosphoproteins induced by both Pvr and InR, which comprise signaling mediators for common functions in cell survival and proliferation. At the same time, we distinguished Pvr- or InR-associated targets that may mediate receptor-specific functions. A common set of phosphorylation targets for Pvr and InR, either direct or indirect, can be inferred from the reciprocal effects of Pvr knockdown and InR stimulation, comparing ‘low Pvr’ and ‘high InR’ conditions (Fig. 7B; 153 phosphosites: S7 Table). Examples include phosphorylation of Structure specific recognition protein (Ssrp), La related protein (Larp), eukaryotic translation initiation factor 4G (eIF4G), Lamin, NAT1, Claspin, Gartenzwerg (Garz), Nedd4, Nopp140, Lk6, Yorkie (Yki), Stat92E, and Moleskin (Msk). Many of these common signaling mediators function in cell survival and cell proliferation. For example, the transcription factor Yki coordinates cell proliferation and apoptosis by directing the expression of cell cycle and cell death regulators [64]. Stat92E loss-of-function has been reported to inhibit hemocyte proliferation [65,66], while the importin Msk localizes MAP kinase to the nucleus to promote cell proliferation and survival [67]. We found enrichment for the regulation of phosphorylation of components of specific complexes by both InR and Pvr, including the Chs5p/Arf1-binding protein complex, the chromatin remodeling FACT complex, the translation initiation factor 2 complex, the cohesion-Sa complex, TRAPP complex and splicing associated factor complex (Fig. 7D, E). While we do not expect that all components of an individual complex require an alteration in phosphorylation in order for complex activity to change, more confidence for implication of that complex downstream of Pvr or InR is gleaned from multiple components exhibiting altered phosphorylation. As such, we expect that these complexes play key roles downstream of both InR and Pvr.

To distinguish Pvr- or InR-specific targets that may mediate receptor-specific functions we compared phosphoproteomes under ‘high Pvr, low InR’ and ‘low Pvr, high InR’ conditions (S8 Table). Among the Pvr-specific phosphorylations, we identified phosphoproteins involved in cell migration, cytoskeleton, and regulation of cell shape such as CIN85 and CD2AP ortholog (Cindr), Tenascin major (Ten-m), Vacuolar protein sorting 4 (Vps4), Rab7, Rho GTPase activating protein at 15B (RhoGAP15B), and Sprouty (Sty). Cindr is a recognized component of the CIN85 complex, one of three complexes for which multiple components exhibited a dependence on Pvr for specific phosphorylation (Fig. 7F). With respect to InR-specific phosphorylations, we detected phosphoproteins associated with the Gene Ontology Consortium terms growth regulation (i.e. Gp150, Foxo, L, Chico), glycogen metabolism (i.e. Glycogen Synthase), and the innate immune response (i.e. G protein-coupled receptor kinase interacting ArfGAP and Mustard). These differential phosphorylations likely provide receptor specificity and function to modulate the activity of specific complexes such as those over-represented in terms of the number of components modulated by InR activity (Fig. 7G).

## Discussion

**Fig 8:**
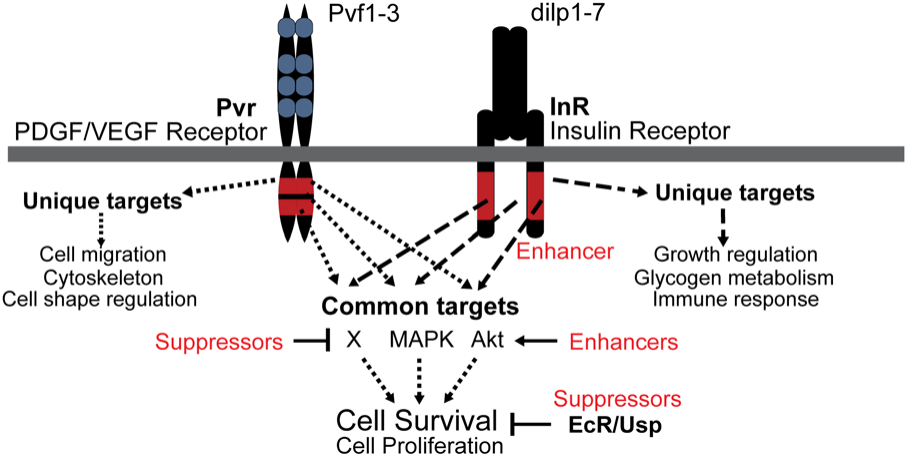
Model of Pvr and InR impact on cell survival control. Signaling scheme of Pvr, and its Enhancer InR and Suppressors EcR/Usp, which were identified by RNAi screening. Pvr and InR trigger the phosphorylation of many common targets of redundant survival pathways (see also Fig. 1F). In addition, each receptor generates specific outputs as a consequence of unique substrate targeting. Enriched Gene Ontology Consortium terms are indicated. EcR and Usp function through a different mode that is largely independent of phosphorylation and Pvr signaling, but rather involves transcriptional regulation of target genes. *Pvr* Suppressors and Enhancers that affect Pvr downstream pathways are illustrated.

Here, we present a genome- and proteome-wide survey in *Drosophila* to identify signaling networks and cellular regulators that control cell survival and cell number. Starting from a genome-wide RNAi screen for modifiers of cell number under *Pvr* sensitized conditions, we established a new proapoptotic role for the EcR complex, and an anti-apoptotic function for InR, in the balance of blood cell number in the *Drosophila* embryo. Phosphoproteomic analyses of *Pvr* deficient cells under low and high InR signaling states enabled the identification of common Pvr and InR phosphorylation targets regulating cell survival, and receptor-specific phosphorylation targets that mediate unique functions of Pvr and InR (model, Fig. 8). Our study further highlights the ability of signaling receptors to modulate their targets depending on cellular context, in our case, specifically based on the activity of other RTKs. These observations are important in light of mechanisms of acquired RTK inhibitor resistance that were recently described [68].

### A sensitized screen to identify novel regulators of cell number

Previously, we demonstrated that Pvr mediates cell survival in the *Drosophila* embryonic hematopoietic system and in *Drosophila* Kc cells in culture [12]. Similar roles for Pvr in other cell populations such as glia were subsequently reported [69]. Here, we find that Pvr also contributes to the proliferation of Kc cells, which is revealed when Pvr-dependent cell death is suppressed. These Pvr functions are well conserved with mammalian systems, where PDGF/VEGF Receptors mediate cell survival and proliferation during normal development [70,71] and in pathologies such as leukemias and other forms of cancer [26,72,73].

Our findings encompassing the role of Pvr in the activation of the Mek/Erk and Akt/Tor pathways are consistent with previous reports of Pvr-dependent phosphorylation of Erk [21,23], the activation of the TOR1 Complex and Erk by Pvr [14], and the physical interaction of Pvr with PVRAP, Grb2, Shc, and the regulatory subunit of PI3K in cell culture [14,74]. Since our screen was designed to eliminate general regulators of cell number and instead focus on those genes that show differential effects under sensitized conditions, it predominantly revealed genes with tumor suppressor-like activities (Pvr Suppressors), many of which were not detected in conventional RNAi screens for cell proliferation or survival previously [17,75–78]. Several of the identified *Pvr* Enhancers (5/14) and half of the *Pvr* Suppressors scored as hits in other genome-wide RNAi screens examining RTK signaling, specifically InR and EGFR signaling using the same screening platform and dsRNA libraries [79]; see S3 Table for specific overlap).

Many genes identified in the screen regulate redundant pro-survival pathways downstream of Pvr (Fig. 8), as was predicted by our initial screening hypothesis (Fig. 1F), and which is also supported by others [14]. However, some regulators identified in the screen instead act in pathways parallel to Pvr signaling, as we demonstrated for InR and EcR signaling. Among the RNAi screen hits, we distinguished three major classes of modifiers. First, we identified a large group of ‘Upstream Genes’ that specifically affect cell number only in signaling competent, but not *Pvr* depleted cells. Among these, we found a large number of ribosomal protein genes. Interestingly, a recent *Drosophila* in vivo study identified ribosomal protein RpS8 as functional upstream regulator of Pvr in hemocytes of the lymph gland, proposing it may exert its function by interaction with Bip1 (bric à brac interacting protein 1), which shows similar phenotypes [16]. While our screen did not identify Bip1, it revealed RpS8 as putative Pvr ‘Upstream Gene’. Ribosomal subunits may promote Pvr expression also as part of the general translation machinery, or may play more specialized roles in translation regulation, according to previous reports on target-specific ribosomal activities that may influence the cellular signaling makeup in development and tumorigenesis [80–82]

Second, inherent to our system, our screen yielded relatively few *Pvr* Enhancers. From this group we chose InR for verification analysis by in vivo genetics, which we further complemented with a phosphoproteomic survey that illuminated synergy between Pvr and InR. Analogous synergistic relationships between InR with other RTKs have been reported in *Drosophila* development [69,83,84], and vertebrate signaling [85]. The specificity of redundant RTK signaling pathways is of major interest is the fields of cell signaling and cancer research and subject of ongoing intense study [68,86].

Third, the screen yielded a group of *Pvr* Suppressors, which function as tumor suppressor-like genes whose loss rescues cell survival under sensitized conditions. This group contains all negative regulators of the Akt/Tor pathway, many of which are known tumor suppressors in mammalian systems [39–41], and several negative regulators of the Mek/Erk pathway such as *mts* and *wdb*, encoding for components of the PP2a complex [42,43], and *Mkp3*, which encodes for a phosphatase known to negatively regulate Erk [44]. As expected, several genes identified in the *Pvr* modifier screen also scored in previous screens for signaling mediators of the Pvr, Akt/Tor and RTK/Erk pathways [14,83,87]. The screen also revealed novel, or only recently characterized, genes. CG6182 is an ortholog of the mammalian TBC7, that interacts physically with Tsc1 [88]; GckIII is a counterpart of mammalian Serine Threonine Kinase 25 (STK25), also known as SOK1, that localizes to the Golgi [89] and induces cell death upon overexpression in mammalian cell culture [90]. Some of the identified genes have been characterized in *Drosophila*, yet no role in cell number control in the embryo has been described. For example, we identified multiple members of the Brahma SWI2/SNF2 family ATPase chromatin-remodeling complex [46,47], with *osa* and *dalao* scoring as *Pvr* Suppressors, and *Brahma associated protein 60kD* (*Bap60*) and *moira* (*mor*) scoring in mixed categories. Two of the strongest hits among the *Pvr* Suppressors were genes encoding the nuclear hormone receptors EcR and Usp [27,45], which we followed up with subsequent analyses.

### Role of the EcR complex in embryonic cell death

EcR and Usp have previously been studied for their roles in proliferation, differentiation and cell death during larval molting and metamorphosis [50,61,91]. In Kc cells, the EcR/Usp ligand ecdysone has been known to arrest the cell cycle and trigger a cell differentiation program [52–54]. However, neither in the embryo nor in Kc cells has ecdysone signaling been previously associated with cell death [51,92]. Here, we describe a role for ecdysone signaling in embryonic cell death, a function revealed only under sensitized conditions or when directly stimulating EcR pathway activity. When treating Kc cells with 20E, we find that the EcR targets *E93* and *rpr* are transcriptionally upregulated, consistent with previous reports describing these genes as transcriptional targets of EcR [93]. E93 and Rpr drive apoptosis [57,94–96] and are required for ecdysone-induced death of the larval midgut and salivary glands during metamorphosis and in the larval cell line l(2)mbn [56,57,97–100]. As for the mechanism of cell death rescue by *EcR* silencing, we were unable to detected measurable levels of *Halloween* gene expression, which is required for biosynthetic maturation of 20E [101]. We therefore propose that Kc cultures produce low levels of 20E through low-level expression of Halloween genes, or the EcR complex may have residual pro-death functions even in the absence of ligand. Previous publications have suggested that the unligated EcR complex has an active role and can bind to ecdysone response elements [102,103].

Vertebrate counterparts of EcR and Usp are the liver X receptors (LXRs), and retinoid X receptor (RXR), respectively [104,105]. RXR plays central roles in cell proliferation, apoptosis, and differentiation [106–108] during development and in pathologies such as cancer and metabolic disease [109,110]. Lack of activation of the RXR/Retinoic acid receptor (RAR) pathways causes Acute Promyelocytic Leukemia (APL) and other malignancies due to impairments in cell differentiation and increased cell survival [109,111,112] and treatment with synthetic retinoids or rexinoids has proven promising in reverting malignant phenotypes [109,111]. Interestingly, dependence on additional anti-apoptotic pathways has been reported in RxR-dependent APL. In particular, Akt/Tor signaling contributes to the increased cell survival in APL, and, consequently, dual therapy with PI3K inhibitors and retinoids has shown great therapeutic promise [113].

### Signaling by InR and the EcR complex

*Drosophila* InR and Akt/TOR signaling were recently reported in several studies for their multifaceted roles in the regulation of lymph gland hemocytes, an independent blood cell lineage in *Drosophila* [114–116]. *Drosophila* InR further promotes the trophic survival of germline stem cells in the *Drosophila* ovary [117], which relies on its downstream mediator Tor [118]. Besides this, *Drosophila* InR is known mostly for its role in cell growth and regulation of body and organ size [119]. In contrast, the related mammalian Insulin-like Growth Factors 1 and 2 (IGF1 and IGF2) play important roles in the trophic survival of various cell types [120]. Our study now demonstrates that *Drosophila* InR also exerts trophic function in embryonic blood cells, which is revealed once redundant receptor activity such as Pvr activity is suppressed.

Several mammalian orthologs of InR phosphorylation targets uncovered from our phosphoproteomic analyses have been previously reported to be regulated by insulin, such as Ssrp, Larp, eIF4G, Lamin, NAT1, Claspin, Garz, Nedd4, Nopp140, Yki and Lk6 [121,122]. These phosphoproteins are also regulated by Pvr and contribute to the roster of common RTK targets that likely account for Pvr/InR-induced cell survival. An additional example of a commonly targeted phosphosite is the activating phosphorylation of Stat92E [123], a proposed target of the insulin receptor [124]. These examples highlight the success of our phosphoproteomic approach to uncover *bona fide* targets shared by InR and PVR. The approach also unveiled novel downstream effectors. For example, the requirement of Pvr for Ssrp phosphorylation hints to a relationship between Pvr and the chromatin remodeling FACT complex, a heterodimer comprised of Ssrp and Dre4 [125]. This hypothesis is reinforced by 1) the suppression of *Pvr* deficient cell proliferation by *dre4* knockdown; and 2) a reported two-hybrid interaction between Pvr and Spt6 [126], a component of an elongation complex that includes FACT [127]. The possibility that FACT functions downstream of these RTKs to regulate transcriptional initiation and elongation as a cell survival mechanism will be an interesting area of future investigation.

Our phosphoproteomics experiments additionally uncovered InR-specific phosphorylations: *e*.*g*. phosphosites on Foxo, Unkempt (Unk), Chico, Tsc1, Spaghetti (Spag), L, Ajuba (Jub), and Git. Notably, many of these proteins were identified by affinity purification and mass spectrometry as components of an InR/Tor protein interaction network [128], supporting our proposition that indeed these phosphoproteins serve InR-specific functions. Remarkably, four of the thirty high confidence *Pvr* Suppressors and two of the fourteen high confidence *Pvr* Enhancers exhibited altered phosphorylation specifically under InR activation indicating these localized phosphorylation events may be critical for the rescue of *Pvr* deficient cells provided by InR stimulation.

Our analysis identified very few *EcR*-dependent phosphoproteins, however, we cannot rule out that these few may indeed regulate cell number. For example, phosphorylation of the methionine sulfoxide reductase Ecdysone-induced protein 28/29kD (Eip71CD) was upregulated by *EcR* knockdown. Eip71CD confers protection to oxidative stress, increases cell size and number, and promotes longevity [129,130]. Additionally, phosphorylation of the diacylglycerol O-acyltransferase Midway (Mdy) was upregulated upon *EcR* knockdown. *mdy* mutant egg chambers exhibit premature nurse cell death and degeneration during mid-oogenesis [131] comparable to *EcR* and *Eip75B* germline clones [132]. We observed an upregulation of phosphorylated Transforming acidic coiled-coil (Tacc) in *Pvr* deficient cells subjected to either *EcR* knockdown. Vertebrate TACC proteins interact with RxRβ to regulate specific gene expression [133]. Phosphorylation could potentially influence Tacc interaction with Usp, the *Drosophila* ortholog of RxR, and consequently impact Usp-dependent gene expression, thereby permitting cell survival. We cannot exclude the possibility that, due to incomplete coverage, our phosphoproteomics analyses may have failed to capture critical phosphorylation changes induced by *EcR* knockdown that account for *Pvr* deficient cell survival. Our analyses did, however, generate a list of candidates for future study and highlight the substantially different responses by insulin and *EcR* knockdown to rescue *Pvr* loss.

### Signaling networks in mammalian development and disease

The dual dependence of *Drosophila* Kc cells and embryonic hemocytes on the Akt/TOR and Mek/Erk pathways (this study and [12,14] echoes the dependence of many mammalian cells, in particular tumor cells, on these two signaling pathways. In addition to concomitant activation by upstream receptors, Akt/Tor and Mek/Erk signaling further show a substantial degree of crosstalk between each other [134]. Dual inhibition of these two pathways has therefore become a promising approach in targeted cancer therapies [135]. However, many molecularly targeted approaches remain challenging due to the plasticity of signaling, the involvement of additional undefined redundant signaling pathways, and the variation of signaling networks downstream of even closely related receptors [68,135]. Findings from the *Drosophila* model provide insight into the pools of common and unique signaling targets of the related RTKs Pvr and InR. Further, this study suggests that the qualitative signaling specificity of receptors can be switched in response to the signaling status of the cell. This notion may be of wide-reaching consequences for many cellular processes, and requires careful consideration when aiming for the experimental or therapeutic manipulation of signaling systems.

## Materials and Methods

### Fly stocks and crosses

Fly lines used were: *Pvr* ^*1*^/CyO [12], *srpHemo*GAL4 [12], *Pxn-GAL4* [136], UAS-*PvrΔC* [12], *UAS*-*p35* [30], UAS-*srcEGFP* (E. Spana), UAS-*lacZ*nls (E. Spana), UAS-m*CD8*::*GFP* [137], UAS-Stinger [138], UAS-*EcRA*, UAS-*EcRB1*, UAS-*EcR*B2 [57], UAS-*EcR*B1 W650A (dominant-negative) [59] and UAS-*EcR* A W650A (dominant-negative) [60], UAS-*InR*-dn [139].

For in vivo quantification of hemocytes in *Drosophila* embryos, the *srp-Hemo-GAL4* driver was used to express UAS-lacZnls and UAS-*srcEGFP* in hemocytes. Genotypes of *Pvr* ^*1*^ mutant rescue/enhancement experiments were: *Pvr* ^*1*^,UAS-*srcEGFP*/ *Pvr* ^*1*^,*srpHemo*GAL4; UAS-*p35*/UAS-*lacZ*nls and *Pvr* ^*1*^,UAS-*EcR* (A or B1) W650A or UAS-*InR*-dn/*Pvr* ^*1*^,*srpHemo*GAL4; UAS-m*CD8*::*GFP*/UAS-*lacZ*nls. Genotype for the alternative rescue/enhancement of Pvr dominant-negative expressing hemocytes were: *srpHemo*GAL4, UAS-*srcEGFP*/+; UAS-*PvrΔC*, UAS-*EcRA* W650A or UAS-*InR*-dn/ UAS-*lacZ*nls.

### Embryo staining, stimulation, and microscopy

Embryos were collected on apple juice agar plates and fixed and stained as described previously [12]. Antibodies used were goat anti-GFP (1:1500) (Molecular Probes) and mouse anti-β-Gal (1:750) (Promega), and Alexa Fluor secondary antibodies (Invitrogen) Imaging was done on Leica DMI 4000B and Leica SP5 microscopes. Hemocyte counts were conducted under fluorescent microscopy at 40X, assessing 10 independent embryos per genotype and stage. Standard deviations and p values by Student’s t-test were calculated.

### Live hemocyte counting

To examine hemocyte numbers at the embryo-larva transition, hemocytes were marked by Pxn-GAL4 driven expression of UAS-Stinger. The transgenic driver UAS-Stinger; Pxn-GAL4 was crossed to w1118 (control), or UAS-EcRA dn, respectively. Dechorionated embryos or larvae from 2 hour timed collections were mounted under glass slides and subjected to visual/manual counting under a fluorescence microscope. At least ten embryos or larvae per time point and genotype were assessed. Standard deviations and p values by Student’s t-test were calculated.

### Cell maintenance and stimulation

Kc167 cells [140], here labeled Kc, were cultured in Schneider’s *Drosophila* Medium (Millipore, Gibco) supplemented with 10% Fetal Bovine Serum (FBS) and 1000 units/ml Penicillin and 1000mcg/ml Streptomycin. Insulin was supplemented to a final concentration of 5 μg/ml for InR stimulation experiments.

20E experiments: 20-Hydroxyecdysone (Sigma-Aldrich), 20E, was dissolved in ethanol to make a 5mg/ml stock. A subsequent stock of 1μg/ml stock was made by diluting in water. 1x10^5^ cells were seeded into each well of a 24 well plate and 20E was added to achieve the indicated final concentrations.

### p35 stable cell line

All cell experiments were based on Kc167 cells, in short Kc. Effectene Transfection Reagent (Qiagen) was utilized for transfection. Kc cells were co-transfected with driver Actin-GAL4, UAS-puromycin, UAS-GFP and UAS-p35 plasmid constructs. Three days after transfection, cells were selected with puromycin 10ug/ml. After 2 weeks, surviving cells were harvested and sorted by Fluorescence-activated cell sorting (FACS) to isolate the highest 20 percentile of GFP-expressing cells. To further select cells that are resistant to apoptosis, *thread* RNAi knockdown was used to eliminate cells with weak resistance to caspase-dependent apoptosis. The surviving cells were expanded for experimental use. The presence of p35 transgene in the p35 stable cell pool was confirmed by PCR verification.

### Cell-based RNAi

RNAi knockdown was performed as described previously [141]. Briefly, Kc167 cells were re-suspended and diluted in serum-free medium before seeding. dsRNAs targeting each specific gene were added and incubated for 45 minutes before supplementing with complete medium with FBS to adjust to a final concentration of 10% FBS.

### Genome-wide RNAi screening

We screened a set of 62 384-well plates that were pre-arrayed with dsRNAs, corresponding to 22,914 distinct amplicons based on Flybase release 5.51 of the *Drosophila* genome corresponding to 13,777 unique genes [31], and 7463 Sanger predictions [142] (DRSC). To determine differential effects between *Pvr* silenced and control cells, we screened each plate under two conditions, dsRNA-mediated knockdown of *Pvr*, or knockdown of a control (*GFP*). All experiments were performed in duplicate. Each well contained 0.25ug of pre-arrayed dsRNA. Before seeding, Kc cell suspensions were pre-mixed with *Pvr* or control (*GFP*) dsRNAs in batch, corresponding to a final concentration of 0.3ug per well. Cells were seeded at a density of 7,000 cells/well and incubated for 4 days. CellTiter-Glo assay (Promega) was performed according to the instructions of the manufacturer, and luminescence was read using Analyst GT or SpectraMax plate readers (Molecular Devices). Liquid handling was performed using WellMate (Matrix), MicroFill (BioTek), or MultiDrop (Thermo), high-throughput dispensers. Z scores [z = (χ-μ)/σ] were calculated as follows: μ = Mean of readings from controls wells (*i*.*e*. wells without pre-arrayed candidate dsRNAs), σ = Standard deviation from readings of the control wells. χ = Reading of candidate gene well. Z score for *Pvr* knockdown condition (Z[*Pvr*]) and for control knockdown condition (Z[*GFP*]) were generated and the differential effects in *Pvr* knockdown condition and control knockdown were calculated by the difference of each Z scores (i.e. Zdiff = Z[*Pvr*]- Z[*GFP*]). Cluster analysis of primary screen data was performed of amplicons scoring ZDiff> = 2.0 and ZDiff< = -2.0, using Z[*Pvr*], Z[*GFP*], and Zdiff values for each amplicon. Analysis included hierarchical clustering by centered correlation, and complete linkage, and results were displayed using TreeView [143]. For verification screening, genes were selected at cutoffs of ZDiff> = 2.2 and ZDiff< = -2.2, and one or two amplicons per gene, non-overlapping with the primary screen amplicons, and devoid of 19bp off-target overlaps, were utilized (DRSC). Secondary screening involved differential screening of *Pvr* RNAi and *GFP* RNAi cells as outlined above. Assays were performed in replicate and repeated in two independent duplicates. Final, ‘high confidence’ *Pvr* modifiers were determined by calculation of final average ZDiff scores determined from the averaged ZDiff scores of all amplicons targeting specific genes that were evaluated in both primary and secondary screening. Regarding the error rate of the screen, we generated false positive and false negative rates as follows: To evaluate false positives we i) assembled a list of 355 protein-coding genes that are not expressed across *Drosophila* tissue/stage/cell lines based on both modEncode RNA-Seq data as well as FlyAtlas data; ii) compared this list with genes scoring in the primary screen (there is only 1 gene overlapping and the relevant amplicon has >5 predicted off targets); and iii) estimated a false positive rate: 1/355 = <1%. To evaluate false negatives we i) assembled a list of 38 high confidence genes based on secondary screening hits, which are the genes that scored with at least 2 independent amplicons and each amplicon was consistently scored among replicates; ii) identified all amplicons relevant to these 38 genes from the genome library and found 80 of them. 41 scored in the primary screen while 39 failed to score; and iii) used these numbers to calculate a false negative rate: 39/80 = 49%.

### Cell counting, EdU and TUNEL assays

To obtain cell counts, 1x10^5^ cells were seeded into each well of a 24-well plate followed by treatment with dsRNAs or 20E. 3.3ug of dsRNA targeting each specific gene knockdown was added. After culturing for the indicated period of time, cells were re-suspended and diluted 1:1 with 0.4% Trypan Blue. Numbers of viable/dead cells were assessed by hemocytometer counting based on Trypan Blue exclusion/staining.

For EdU and TUNEL assays, 20,000 Kc cells were seeded into each well of 96-well black clear bottom plate and immediately treated with dsRNAs or 20E. 0.825ug of dsRNA was used to target each specific gene knockdown.

To assess cell proliferation, cells were incubated for 4 hours with 10uM of Click-iT EdU (Invitrogen) one or several days after the dsRNAs or 20E treatment. The Click-iT EdU cell proliferation assay was conducted according to manufacturer's instructions.

For assessing cell death, cells were processed by TUNEL assay according to manufacturer's instructions (Invitrogen).

Stained cells were counted visually/manually and by ImageJ automated cell quantification. In brief, for ImageJ analysis, still images were converted to 8 bit images and cells were selected by setting a threshold against the background. Highlighted cells were then counted by the ‘Analyze Particles’ function. At least three still images for each sample were taken at random sites using a 40X objective. Percentages of EdU or TUNEL positive cells were calculated as follows: (# of EdU or TUNEL positive cells/ total # of cells) * 100. Cell culture figures show compilations of three independent biological replicate experiments. Error bars indicate standard deviation. Student’s t-test as indicated. * for p < 0.05; ** for p < 0.01; *** for p < 0.001; NS for not significant.

### dsRNAs design and generation

In most cases, dsRNA amplicon sequences were selected by the *Drosophila* RNAi Screening Center (DRSC), as indicated by DRSC amplicon numbers. Primers used for generating the amplicon template contained a 5' T7 RNA polymerase-binding site (TAATACGACTCACTATAGG) following by the amplicon specific sequences. dsRNAs were generated by in vitro transcription using Megascript T7 transcription kit (Ambion). dsRNAs were purified with a RNeasy Mini Kit (Qiagen) and product size confirmed by agarose gel electrophoresis. dsRNA concentrations were measured with a Nanodrop 2000C spectrophotometer (Thermo Scientific).

### Real time PCR

Total RNA was extracted using a RNeasy mini kit (Qiagen), according to manufacturer's instructions. Total purified RNA were measured with a Nanodrop 2000C spectrophotometer (Thermo Scientific). 1ug—0.1ug of purified RNA was reversed transcribed into cDNA using an iScript cDNA synthesis kit (Biorad). Real time PCR reactions were carried out using iQ SYBR Green Supermix (Bio-Rad) on a Bio-Rad CFX96 Real Time System and gene expression levels were analyzed with CFX Manager Software (Bio-Rad). Primers for real time PCR assays were designed using web-based software ProbeFinder (Roche Applied Science Universal ProbeLibrary Assay Design Center) or by the author. Primer sequences for real-time PCR assessment will be made available upon request.

### Protein lysates and immunoblotting

Kc cells were lysed using Triton lysis buffer (50mM Tris-HCl (pH 7.5), 150mM NaCl, 1% Trition X-100, 30mM NaF) freshly supplemented with 1mM Na_3_VO_4_ and protease inhibitors (Complete, Roche) and immunoblot analysis was performed as described previously [12]. Primary antibodies were obtained from Cell Signaling Technology except monoclonal anti-β-tubulin (Sigma T5168), anti-Pvr [12], anti-EcR (Developmental Studies Hybridoma Bank, DSHB) and anti-histone H3 (Abcam 39950); signal was detected by HRP conjugated secondary antibodies (Amersham NA934V/NXA931 and Jackson ImmunoResearch 706–035–148) and ECL.

### Sample preparation for mass spectrometric analysis

Kc cells were serum starved for 1 hr, incubated with dsRNA for 30 minutes and then diluted either in Schneider’s *Drosophila* Medium (Gibco) supplemented with Fetal Bovine Serum (FBS) (final concentration of 10%), Penicillin (50 units/ml final concentration), and Streptomycin (50 ug/ml final concentration), with or without insulin (5ug/ml final concentration). After two days cells were lysed in: 8M urea, 75mM NaCl, 50mM Tris-HCl pH 8.2, 1mM NaF, 1mM β-glycerophosphate, 1mM sodium orthovanadate, 10mM sodium pyrophosphate, 1mM PMSF, EDTA-free Protease Inhibitor Cocktail Tablet (Roche). One milligram of protein from each sample was reduced with 5mM dithiothreitol at 56°C for 25 minutes. Cysteines were alkylated with 14mM iodoacetamide for 30 minutes at room temperature in the dark. Unreacted iodoacetamide was quenched by incubation with additional dithiothreitol to 5mM for 15 minutes at room temperature in the dark. Lysates were diluted 1:5 with 25mM Tris-HCl, pH 8.2 and CaCl_2_ added to 1mM. Digestion with 5ug sequencing grade trypsin (Promega) was overnight at 37°C with agitation. Peptides were acidified with 10% trifluoroacetic acid and desalted using 1cc Sep-Pak tC18 solid-phase extraction cartridges (Waters). Eluted peptides were lyophilized, resuspended in 200mM Na-HEPES pH8.2, and labeled with TMT reagent (Thermo Scientific) in anhydrous acetonitrile (2mg TMT reagent per sample) for 1 hour at room temperature. TMT labeling was as follows:

**Experiment 1**. control dsRNA, biological replicate #1: 126 (high Pvr, low InR); control dsRNA, biological replicate #2: 127 (high Pvr, low InR); control dsRNA + insulin, biological replicate #1: 128 (high Pvr, high InR); control dsRNA + insulin, biological replicate #2: 129 (high Pvr, high InR); control dsRNA + EcR dsRNA, biological replicate #1: 130 (high Pvr, low EcR); control dsRNA + EcR dsRNA, biological replicate #2: 131 (high Pvr, low EcR).

**Experiment 2.** *Pvr* dsRNA + control dsRNA, biological replicate #1: 126 (low Pvr, low InR); *Pvr* dsRNA + control dsRNA, biological replicate #2: 127 (low Pvr, low InR); *Pvr* dsRNA + control dsRNA + insulin, biological replicate #1: 128 (low Pvr, high InR); *Pvr* dsRNA + control dsRNA + insulin, biological replicate #2: 129 (low Pvr, high InR); *Pvr* dsRNA + EcR dsRNA, biological replicate #1: 130 (low Pvr, low EcR); *Pvr* dsRNA + EcR dsRNA, biological replicate #2: 131 (low Pvr, low EcR).

**Experiment 3**. *Pvr* dsRNA + control dsRNA: 126 (low Pvr, low InR); *Pvr* dsRNA + control dsRNA + insulin: 127 (low Pvr, high InR); *Pvr* dsRNA + EcR dsRNA: 128 (low Pvr, low EcR); control dsRNA: 129 (high Pvr, low InR); control dsRNA + insulin: 130 (high Pvr, high InR); control dsRNA + EcR dsRNA: 131 (high Pvr, low EcR).

Reactions were quenched by the addition of hydroxylamine to 0.3% and incubation at room temperature for 15 min. Labeled peptides were combined, lyophilized, and stored at -80°C until further processing. Samples were acidified with 10% trifluoroacetic acid and desalted using a 3cc Sep-Pak tC18 solid-phase extraction cartridge (Waters). Phosphopeptides were enriched by strong cation exchange chromatography (SCX; [144]). Lyophilized peptides were resuspended in 400 ul SCX buffer A (7 mM KH2PO4, pH 2.65, 30% acetonitrile) and injected onto a SCX column (Polysulfoethyl aspartamide, 9.4 mm×250mm, 5 uM particle size, 200 Ǻ pore size, PolyLC). A gradient was developed over 35 min from 0% to 30% buffer B (7 mM KH2PO4, pH 2.65, 30% acetonitrile, 350 mM KCl) at a flow rate of 2.5 ml/min. 12 fractions were collected and lyophilized. Peptides were then desalted with 1cc Waters Sep-Pak tC18 solid-phase extraction cartridges and subjected to TiO_2_ based phosphopeptide enrichment [145] using 0.5mg titanium dioxide microspheres per mg protein. Eluates were further desalted using STAGE tips [146] and lyophilized. Samples were reconstituted in 4ul 5% formic acid / 5% acetonitrile.

### Mass spectrometric analysis

In most signaling systems, the major gatekeeper of signal transduction is protein phosphorylation, which can be adjusted rapidly according to the needs of a cell. A caveat of solely measuring phosphorylation is that a change in phosphopeptide levels for any particular peptide can result from a change in phosphorylation of the peptide, or from a change in levels of that protein. We expect that roughly a quarter of altered phosphorylation we observe would be explained by the latter mechanism, based on previous reports (Bodenmiller et al. Science Signaling 2010 and Wu et al. Mol. Cell Proteomics, 2011; Sopko et al. Dev Cell 2014). Given that coverage of the *Drosophila* proteome is not yet comprehensive in a single mass spec run, normalization of phosphorylation to protein amounts can only be estimated. Further, lowly expressed proteins are often missed. For these reasons, we chose to focus exclusively on phosphorylation in our study. Samples were subjected to LC-MS/MS with an Orbitrap Velos Pro mass spectrometer (Thermo Scientific) using higher-energy collision dissociation (HCD; [147]) and a top ten method [148]. MS/MS spectra were searched against a composite database of *Drosophila melanogaster* proteins derived from Flybase version 5.23 in both the forward and reverse orientation using the Sequest algorithm [149]. Search parameters included: a precursor mass tolerance of 20 ppm; up to two missed cleavages; static modification of TMT tags on lysine residues and peptide N termini (+229.162932 Da) and +57.021464 Da accounting for carbamidomethylation on Cys; dynamic modification of phosphorylation (+79.966330 Da) on Ser, Thr and Tyr and oxidation (+15.994915 Da) on Met. A target-decoy database search strategy [150] enabled thresholding of the false discovery rate (FDR) for MS/MS spectral assignment at 1%. Correct spectral matches were distinguished from incorrect matches using linear discriminant analysis based on parameters including Xcorr, ΔCn, precursor mass error, peptide length, and charge state [151]. The localizations of individual phosphorylations were assigned using the probability-based AScore algorithm [152] and only phosphosites with AScores greater than 13 (p < 0.05) were considered in our analysis. Moreover, only phosphopeptides with isolation specificity greater than 0.75 were considered for further analysis. Further filtering of the dataset resulted in a final protein FDR of ~2% and a peptide FDR near 0.15%. TMT labeling was >98% efficient. For TMT reporter ion quantification, a 0.03 Da window centered on the expected mass of each reporter ion was monitored and the intensity of the signal closest to the expected mass was recorded. Reporter ion signals were further adjusted to correct for impurities associated with each TMT label, as described elsewhere [153]. Raw TMT reporter ion intensities for individual phosphopeptides were normalized to the summed reporter ion intensity for each TMT label. Adjusted reporter ion intensities were averaged between replicates. Peptides for which only one replicate TMT labeled sample generated detectable reporter ions were excluded from further analysis.

### Database information

Complete information on Pvr modifier screen data, and DRSC library dsRNA amplicons can be accessed at http://www.flyrnai.org/.

## Supporting Information

S1 Fig Immunoblot examining Pvr knockdown efficiency and cell cycle progression in Kc and Kcp35 cell lines. Immunoblot examining Pvr, phospho-histone H3, and total histone H3 after two days with no treatment or two days of *Pvr* or GFP dsRNA treatment. Samples of equal numbers of cells were loaded.
(PDF)

S2 Fig RNAi knockdown efficiencies of amplicons. Summary of dsRNA-mediated knockdown efficiencies assessed by quantitative real time PCR (qRT-PCR) or immunoblot.
(PDF)

S3 Fig Expression of *Pvf2* following *Akt* knockdown. Plotted (y-axis) is the level of *Pvf2* transcript remaining in Kc cells treated with dsRNA targeting *Akt* relative to a dsRNA targeting GFP. For normalization, *Ribosomal protein L32* was used as a reference gene.
(PDF)

S4 Fig Verification screen cell counts of 22 *Pvr* Suppressors. Live/dead cell counting performed after silencing of 22 *Pvr* Suppressors or insulin stimulation in combination with *Pvr*, and compared to *Pvr* and *GFP* (control) knockdown.
(PDF)

S5 Fig 20HE has no effect on Pvr levels. Immunoblot examining Pvr after treatment of Kc and Kcp35 cells with 0.01 ug/ml 20HE for three days.
(PDF)

S6 Fig Expression of *rpr* and *E93* following *Pvr* and *EcR* knockdown. Plotted (y-axis) is the level of *rpr* or *E93* transcript remaining in Kc cells treated with dsRNA targeting *Pvr* or *EcR* relative to a dsRNA targeting GFP. For normalization, *Ribosomal protein L32* was used as a reference gene. Two non-overlapping qPCR primers for each gene were used. No significant changes were observed.
(PDF)

S7 Fig Hemocyte numbers during the embryo-larva transition. Live hemocyte counts of embryos and larvae at the indicated times after egg laying (AEL), grown at 25C. UAS-Stinger; Pxn-GAL4 was crossed to w1118 (control), or UAS-EcRA dn, respectively. Note that around the time of hatching (grey bar), hemocyte numbers have dropped to about 60% of embryonic counts. Hemocyte-specific expression of UAS-EcRA dn does not significantly protect hemocytes from the decline, as indicated for 22h AEL.
(PDF)

S8 Fig Pvr and EcR knockdown efficiency in mass spectrometry samples. Immunoblot confirming knockdown of *Pvr* and *EcR* (top panels) after two days dsRNA treatment in Kc cells used for phosphoproteomic analysis by mass spectrometry.
(PDF)

S1 Table Primary screen scores. Amplicons of primary *Pvr* modifier screen with resulting ZDiff > = 2 or < = -2. *Pvr* Enhancers, Suppressors, and Upstream Regulators are indicated.
(XLS)

S2 Table Verification screen scores. Verification screen amplicons, Z scores of all replicates with resulting ZDiff values. Homologs and Scores predicted using DIOPT—DRSC Integrative Ortholog Prediction Tool (www.flyrnai.org/cgi-bin/DRSC_orthologs.pl)
(XLS)

S3 Table Averaged final scores. Final scores ZDiffFinal resulting from equally averaging all amplicons of a gene in the primary and secondary screen.
(XLS)

S4 Table Phosphoproteomic data under ‘high Pvr conditions’. Phosphosites identified in Kc cells, a condition of unaltered Pvr activity (‘high Pvr’), in combination with *EcR* knockdown (‘low EcR’) or insulin stimulation (‘high InR’). Duplicate samples were examined.
(XLSX)

S5 Table Phosphoproteomic data under ‘low Pvr’ conditions. Phosphosites identified in *Pvr* RNAi cells, in combination with *EcR* knockdown (‘low EcR’) or insulin stimulation (‘high InR’). Duplicate samples were examined.
(XLSX)

S6 Table Phosphoproteomic data comparing ‘high Pvr’ and ‘low Pvr’ conditions. Phosphosites identified in direct comparison of Kc cells treated with control or *Pvr* dsRNAs, in combination with *EcR* knockdown (‘low EcR’) or insulin stimulation (‘high InR’).
(XLSX)

S7 Table Commonly regulated Pvr and InR phosphosites. Phosphosites predicted to be targeted by both Pvr or InR, based on their common directionality of change under conditions of ‘high Pvr, low InR’ and ‘low Pvr, high InR’.
(XLSX)

S8 Table Differentially regulated Pvr and InR phosphosites. Phosphosites predicted to be targeted by either Pvr or InR specifically, based on their directionality of change under ‘high Pvr, low InR’ and ‘low Pvr, high InR’ conditions.
(XLSX)
