# Supplementary figures and images for: A Systems-Level Interrogation Identifies Regulators of Drosophila Blood Cell Number and Survival
Source: PLoS Genet. 2015 Mar 6;11(3):e1005056. doi: 10.1371/journal.pgen.1005056 (PMC4352040; doi:10.1371/journal.pgen.1005056)

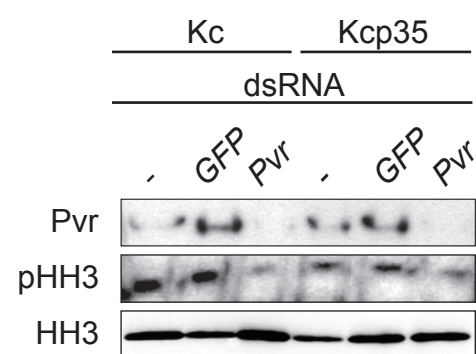

Sopko et al. Suppl. Figure 1

Supplement: S1 Fig — Immunoblot examining Pvr, phospho-histone H3, and total histone H3 after two days with no treatment or two days of Pvr or GFP dsRNA treatment. Samples of equal numbers of cells were loaded. (PDF) [file pgen.1005056.s001.pdf]

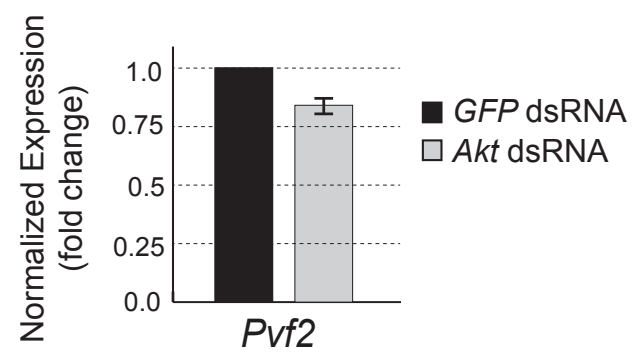

Sopko et al. Suppl. Figure 3

Supplement: S3 Fig — Plotted (y-axis) is the level of Pvf2 transcript remaining in Kc cells treated with dsRNA targeting Akt relative to a dsRNA targeting GFP. For normalization, Ribosomal protein L32 was used as a reference gene. (PDF) [file pgen.1005056.s003.pdf]

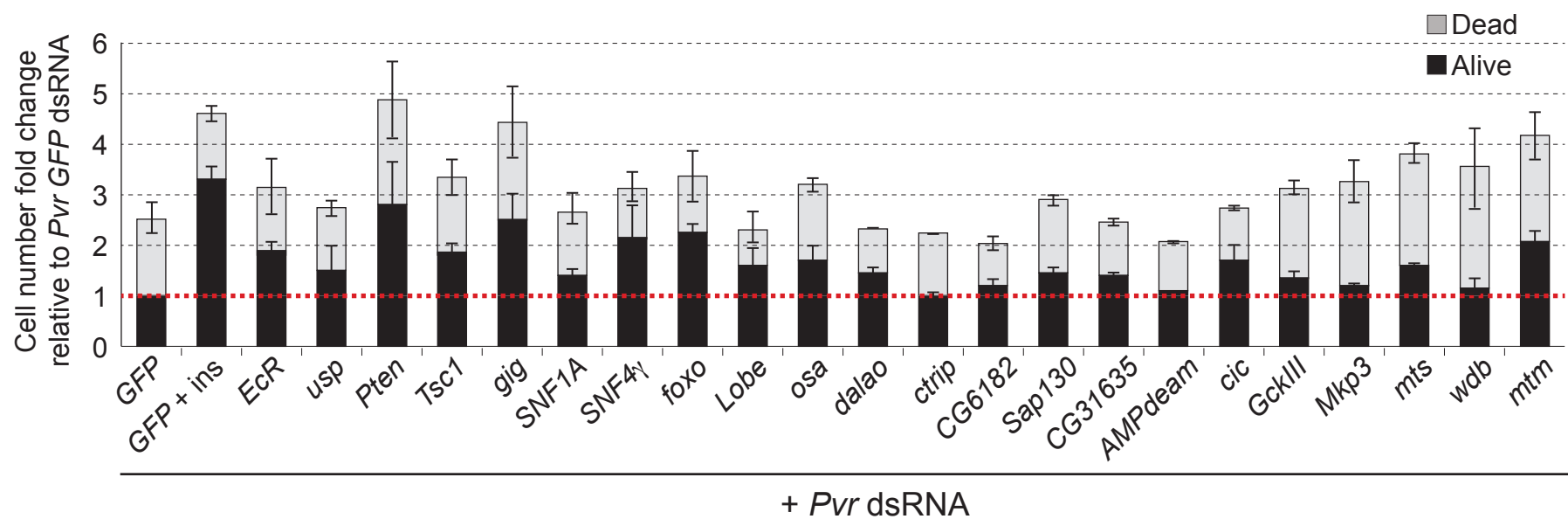

Supplement: S4 Fig — Live/dead cell counting performed after silencing of 22 Pvr Suppressors or insulin stimulation in combination with Pvr, and compared to Pvr and GFP (control) knockdown. (PDF) [file pgen.1005056.s004.pdf]

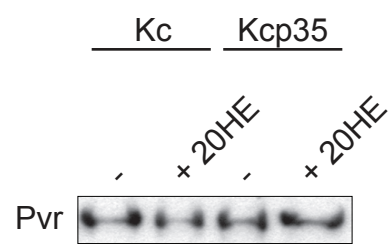

Sopko et al. Suppl. Figure 5

Supplement: S5 Fig — Immunoblot examining Pvr after treatment of Kc and Kcp35 cells with 0.01 ug/ml 20HE for three days. (PDF) [file pgen.1005056.s005.pdf]

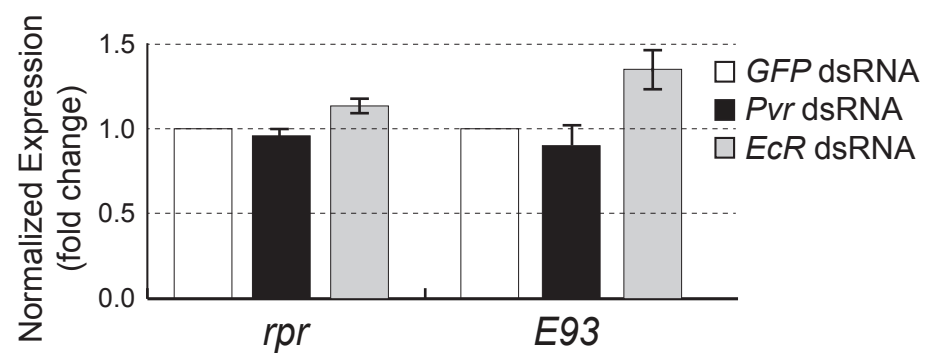

Sopko et al. Suppl. Figure 6

Supplement: S6 Fig — Plotted (y-axis) is the level of rpr or E93 transcript remaining in Kc cells treated with dsRNA targeting Pvr or EcR relative to a dsRNA targeting GFP. For normalization, Ribosomal protein L32 was used as a reference gene. Two non-overlapping qPCR primers for each gene were used. No significant changes were observed. (PDF) [file pgen.1005056.s006.pdf]

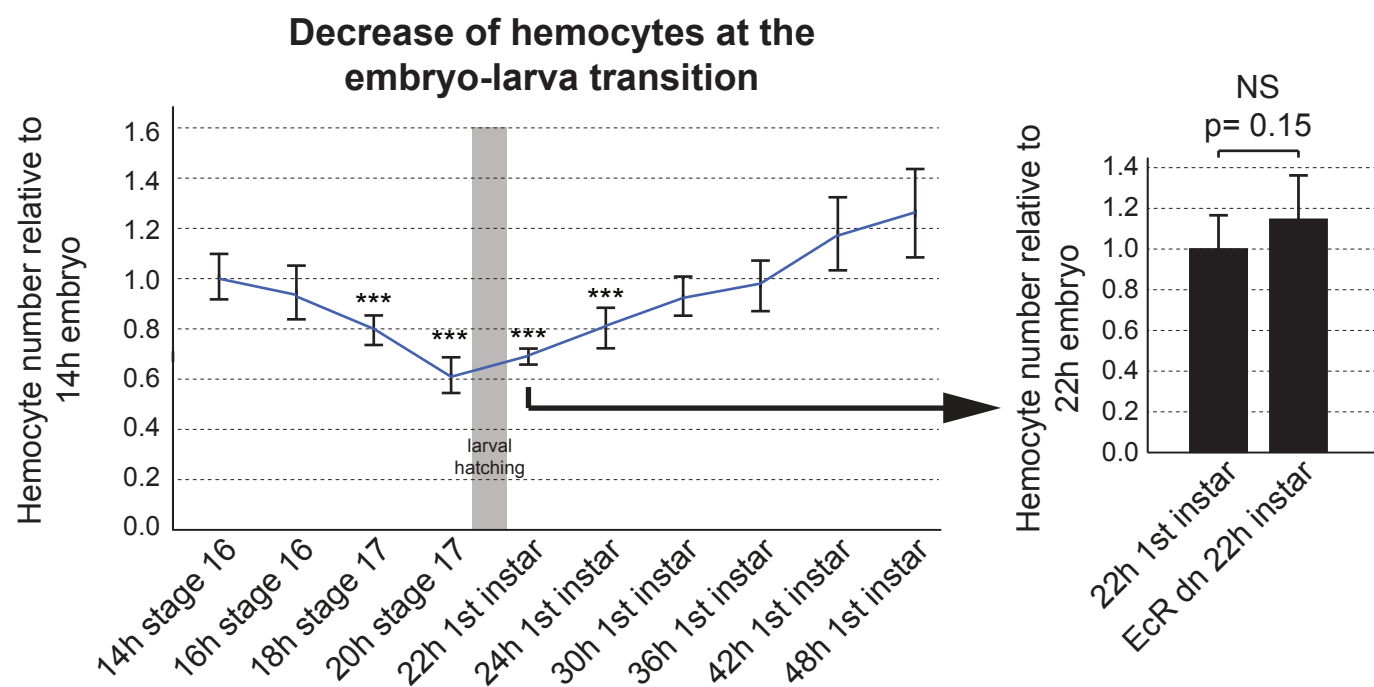

Sopko et al. Suppl. Figure 7

Supplement: S7 Fig — Live hemocyte counts of embryos and larvae at the indicated times after egg laying (AEL), grown at 25C. UAS-Stinger; Pxn-GAL4 was crossed to w1118 (control), or UAS-EcRA dn, respectively. Note that around the time of hatching (grey bar), hemocyte numbers have dropped to about 60% of embryonic counts. Hemocyte-specific expression of UAS-EcRA dn does not significantly protect hemocytes from the decline, as indicated for 22h AEL. (PDF) [file pgen.1005056.s007.pdf]

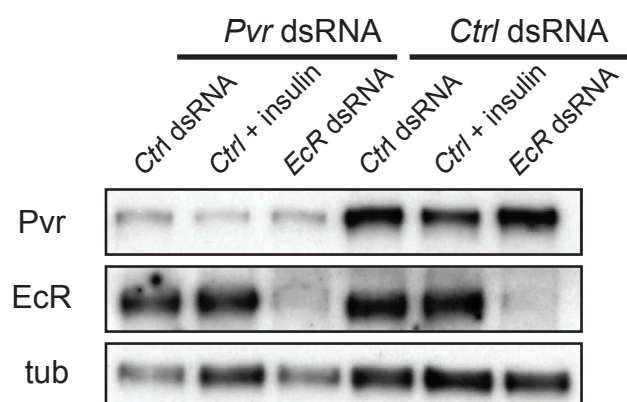

Sopko et al. Suppl. Figure 8

Supplement: S8 Fig — Immunoblot confirming knockdown of Pvr and EcR (top panels) after two days dsRNA treatment in Kc cells used for phosphoproteomic analysis by mass spectrometry. (PDF) [file pgen.1005056.s008.pdf]
