# Supplementary material for: A Systems-Level Interrogation Identifies Regulators of Drosophila Blood Cell Number and Survival
Source: PLoS Genet. 2015 Mar 6;11(3):e1005056. doi: 10.1371/journal.pgen.1005056 (PMC4352040; doi:10.1371/journal.pgen.1005056)
Supplement: S2 Fig — Summary of dsRNA-mediated knockdown efficiencies assessed by quantitative real time PCR (qRT-PCR) or immunoblot. (PDF) [file pgen.1005056.s002.pdf]

Gene	Knockdown Efficiency	Verified by
Pvr	>99%	immunoblot
Akt	>99%	qRT-PCR
Raptor	~80%	qRT-PCR
S6k	~80%	qRT-PCR
Erk	>99%	immunoblot
Mek	>99%	immunoblot
EcR	>99%	immunoblot
usp	~90%	qRT-PCR
Pten	~60%	qRT-PCR
gig	~85%	qRT-PCR

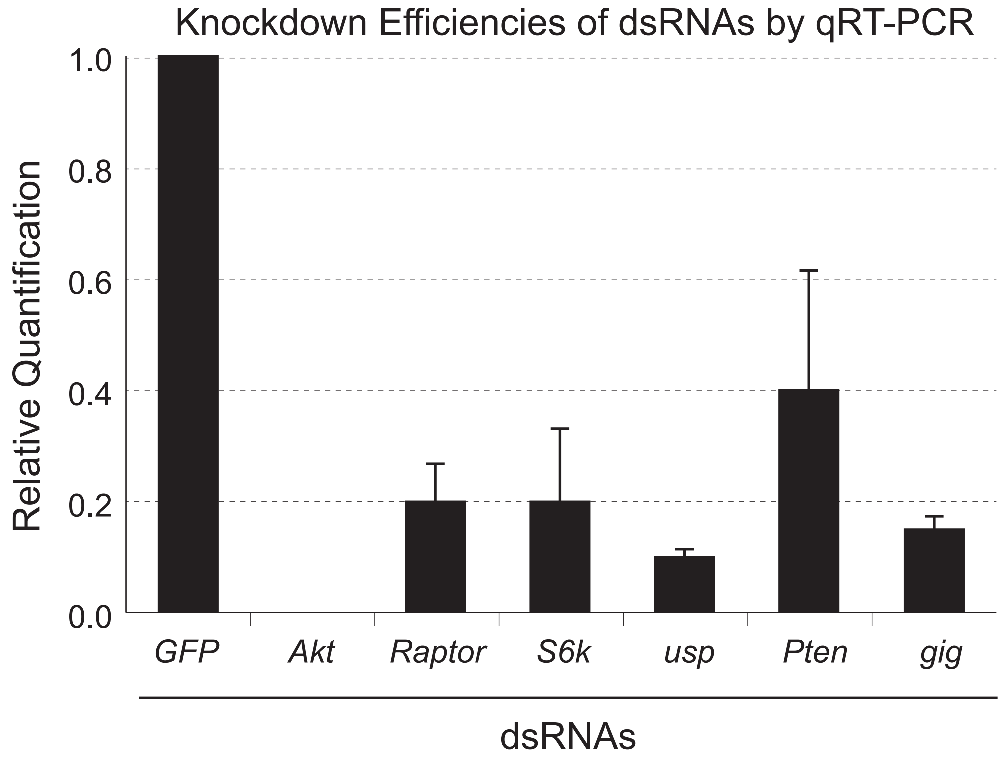

Sopko et al. Suppl. Figure 2
